# Autocrine STAT3 activation in HPV positive cervical cancer through a virus-driven Rac1—NFκB—IL-6 signalling axis

**DOI:** 10.1371/journal.ppat.1007835

**Published:** 2019-06-21

**Authors:** Ethan L. Morgan, Andrew Macdonald

**Affiliations:** School of Molecular and Cellular Biology, Faculty of Biological Sciences and Astbury Centre for Structural Molecular Biology, University of Leeds, Leeds, United Kingdom; Fred Hutchinson Cancer Research Center, UNITED STATES

## Abstract

Persistent human papillomavirus (HPV) infection is the leading cause of cervical cancer. Although the fundamental link between HPV infection and oncogenesis is established, the specific mechanisms of virus-mediated transformation are not fully understood. We previously demonstrated that the HPV encoded E6 protein increases the activity of the proto-oncogenic transcription factor STAT3 in primary human keratinocytes; however, the molecular basis for STAT3 activation in cervical cancer remains unclear. Here, we show that STAT3 phosphorylation in HPV positive cervical cancer cells is mediated primarily via autocrine activation by the pro-inflammatory cytokine Interleukin 6 (IL-6). Antibody-mediated blockade of IL-6 signalling in HPV positive cells inhibits STAT3 phosphorylation, whereas both recombinant IL-6 and conditioned media from HPV positive cells leads to increased STAT3 phosphorylation within HPV negative cervical cancer cells. Interestingly, we demonstrate that activation of the transcription factor NFκB, involving the small GTPase Rac1, is required for IL-6 production and subsequent STAT3 activation. Our data provides new insights into the molecular re-wiring of cancer cells by HPV E6. We reveal that activation of an IL-6 signalling axis drives the autocrine and paracrine phosphorylation of STAT3 within HPV positive cervical cancers cells and that activation of this pathway is essential for cervical cancer cell proliferation and survival. Greater understanding of this pathway provides a potential opportunity for the use of existing clinically approved drugs for the treatment of HPV-mediated cervical cancer.

## Author summary

Persistent infection with HPV is the predominant cause of anogenital and oral cancers. Transformation requires the re-wiring of signalling pathways in infected cells by virus encoded oncoproteins. At this point, a comprehensive understanding of the full range of host pathways necessary for HPV-mediated carcinogenesis is still lacking. In this study we describe a signalling circuit resulting in the aberrant production of the IL-6 cytokine. Mediated by the HPV E6 oncoprotein, it requires activation of the NFκB transcription factor. The autocrine and paracrine actions of IL-6 are essential for STAT3 activation in HPV positive cervical cancers and loss of the pathway results in increased cancer cell death and a reduction in proliferation. This study provides molecular insights into the mechanisms by which a virus encoded oncoprotein activates an oncogenic pathway and identifies potential targets for therapeutic intervention.

## Introduction

Human papillomaviruses (HPV) are a leading cause of squamous cell carcinomas of the ano-genital and oropharyngeal epithelium [1]. High-risk HPVs (HR-HPV), exemplified by HPV16 and 18, are responsible for >99% of cervical, and between 30–70% of oropharyngeal cancers [2]. HR-HPVs encode three oncogenic proteins: E5, E6 and E7, which interact with a multitude of host factors to manipulate signalling pathways necessary for cellular transformation. Whilst the role of the membrane protein E5 in cellular transformation is relatively poorly understood, it has been shown to activate EGFR signalling [3], which is necessary for transformation *in vivo* [4]. EGFR activation is linked to the virus-coded ion channel (viroporin) activity of E5 [5–7]. In contrast, the E6 and E7 oncoproteins have been conclusively shown to play a pivotal role in HPV-mediated transformation [8]. The E7 protein promotes progression of cells through the S phase of the cell cycle via an ability to bind and inactivate pocket protein family members including pRb [9], resulting in release of the E2F transcription factor [10]. Additionally, E7 stimulates the DNA damage response, driving viral replication and genomic instability [11,12]. HPV E6 forms complexes with host E3 ubiquitin ligases and mediates proteasomal degradation of a number of host targets including the p53 tumour suppressor protein, as well as increasing telomerase activity in order to prevent apoptosis and immortalise infected cells [13]. HR-E6 proteins also bind and regulate a selection of PSD95/DLG/ZO-1 (PDZ) domain containing proteins [14,15]. In addition to these classical interactions, emerging evidence shows targeting of additional signalling pathways, including the Wnt and Hippo pathways, contributes to transformation by the HPV oncoproteins [16–18].

The transcription factor signal transducer and activator of transcription (STAT) 3 is an essential regulator of cellular proliferation, differentiation and survival [19]. It is a *bona fide* oncogene and its aberrant activation has been observed in a growing number of malignancies [20]. As such, STAT3 has become an attractive therapeutic target in a diverse range of cancers, including bladder, ovarian and head and neck squamous cell carcinoma (HNSCC) [21].

Oncogenic viruses can activate STAT3 to drive cellular proliferation, necessary for viral replication and tumourigenesis [22]. Using a primary keratinocyte cell culture model, we previously demonstrated that E6 activates STAT3 signalling during the productive HPV18 lifecycle [23]. STAT3 activation was essential for the hyperplasia observed in HPV-containing keratinocyte raft culture models. Increased STAT3 protein expression and phosphorylation also correlated with cervical disease progression in a panel of cytology samples [23]. Although we identified that Janus kinase 2 (JAK2) and MAP kinases were necessary for STAT3 phosphorylation in HPV-containing primary keratinocytes, our understanding of the mechanisms by which E6 mediates this process remains incomplete. Furthermore, inhibition of STAT3 activity in cervical cancer cells results in a profound reduction in cellular proliferation and the induction of apoptosis [24,25], yet the mechanisms underpinning STAT3 activation and function in this scenario remain unknown.

A number of extracellular stimuli including cytokines and growth factors induce STAT3 phosphorylation and signalling [21]. This requires the phosphorylation of tyrosine 705 (Y705) and serine 727 (S727), resulting in STAT3 dimerisation and nuclear translocation, where it is able to regulate gene expression [20]. In particular, members of the IL-6 family of cytokines are key mediators of STAT3 activation through their interactions with the gp130 co-receptor [26].

Here, we show that HPV positive cervical cancer cells have higher levels of phosphorylated STAT3 protein when compared with those that are HPV negative. This results from increased IL-6 production and release, leading to autocrine and paracrine activation of STAT3 via a signalling pathway requiring the IL-6 co-receptor gp130. Mechanistically, we show that IL-6 production is controlled by E6-mediated stimulation of NFκB signalling, which appears to be dependent on an upstream signalling pathway requiring the Rac1 GTPase and the AKT protein kinase. Finally, we demonstrate a correlation between NFκB activation, IL-6 expression and cervical disease progression, suggesting that targeting the IL-6 pathway to prevent STAT3 activation may have therapeutic benefits in cervical cancer.

## Results

### STAT3 protein expression and phosphorylation is increased in HPV positive compared with HPV negative cervical cancer cells

**Fig 1:**
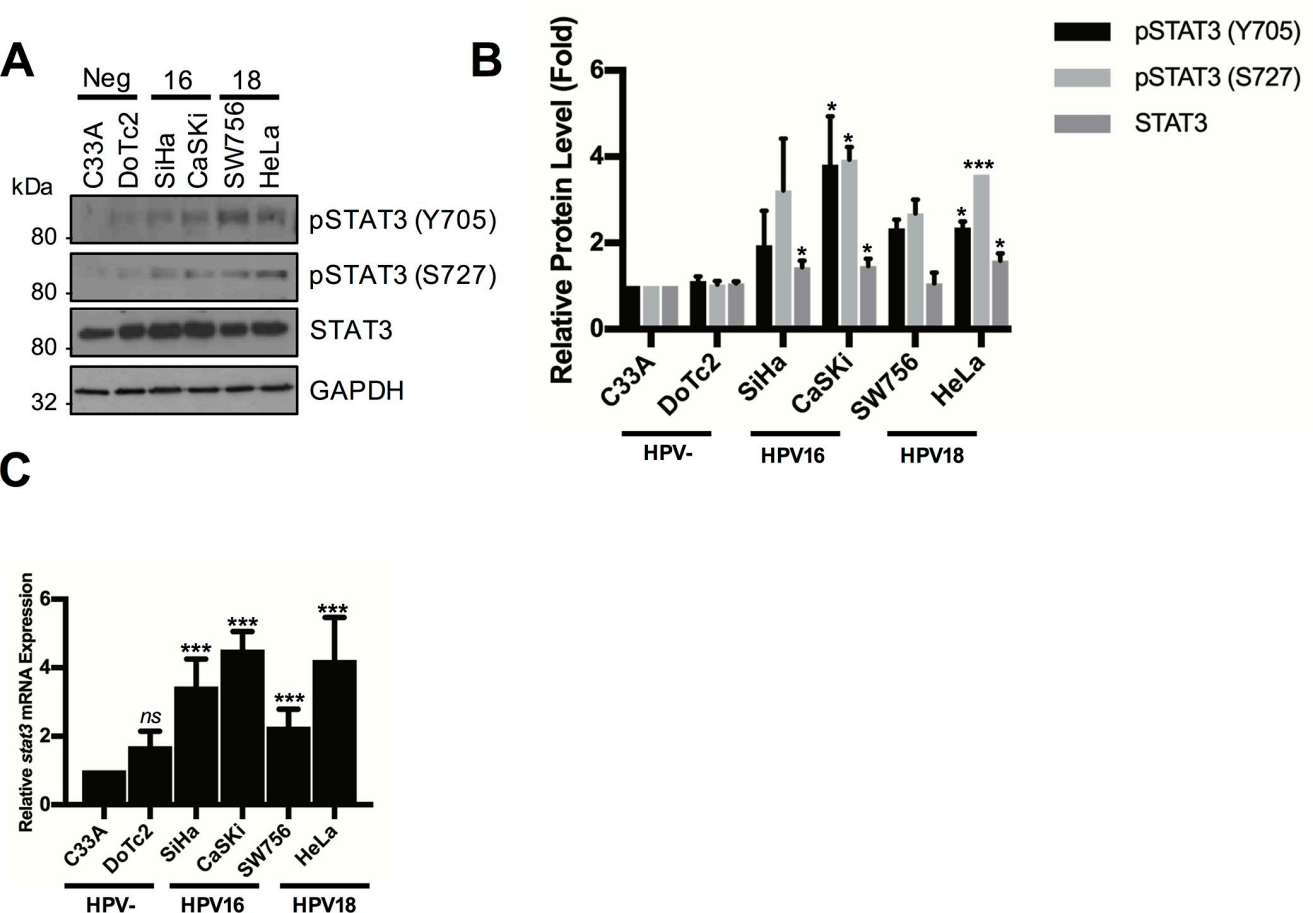
STAT3 phosphorylation is higher in HPV+ verses HPV- cervical cancer cells. **A)** Representative western blot from six cervical cancer cell lines–two HPV- (C33A and Dotc2 4510), two HPV16+ (SiHa and CaSKi) and two HPV18+ (SW756 and HeLa)–for the expression of phosphorylated (Y705 and S727) and total STAT3. GAPDH served as a loading control. Data are representative of at least three biological independent repeats. **B)** Quantification of the protein band intensities in **A)** standardised to GAPDH levels. Bars represent the means ± standard deviation from at least 3 independent biological repeats. *P<0.05, ***P<0.001 (Student’s t-test). **C)** Expression level of STAT3 mRNA in cervical cancer cells measured by RT-qPCR. Samples were normalized against U6 mRNA levels. Representative data are presented relative to the HPV- cervical cancer cells. Bars are the means ± standard deviation from at least three biological repeats. ***P<0.001 (Student’s t-test).

We analysed the level of STAT3 phosphorylation in a panel of six cervical cancer cell lines to establish whether HPV augmentation of STAT3 phosphorylation was evident. The panel included two HPV negative (HPV-; C33A and DoTc2), two HPV16 positive (HPV16+; SiHa and CaSKi) and two HPV18 positive (HPV18+; SW756 and HeLa) lines. Both HPV16+ and HPV18+ cancer cells retained markedly higher levels of STAT3 phosphorylation at both Y705 and S727 residues compared to the HPV negative cell lines (Fig 1A and 1B). The overall abundance of STAT3 protein was also increased in the HPV positive compared with HPV negative cervical cancer cells (Fig 1A and 1B) and this correlated with an increase in the levels of STAT3 mRNA expression in HPV positive compared to the HPV negative cell lines (Fig 1C). Together, these data demonstrate increased levels of STAT3 expression and phosphorylation in HPV positive cervical cancer cell lines.

### A secreted factor in the media of HPV positive cells can induce STAT3 phosphorylation in HPV negative cervical cancer cells

**Fig 2:**
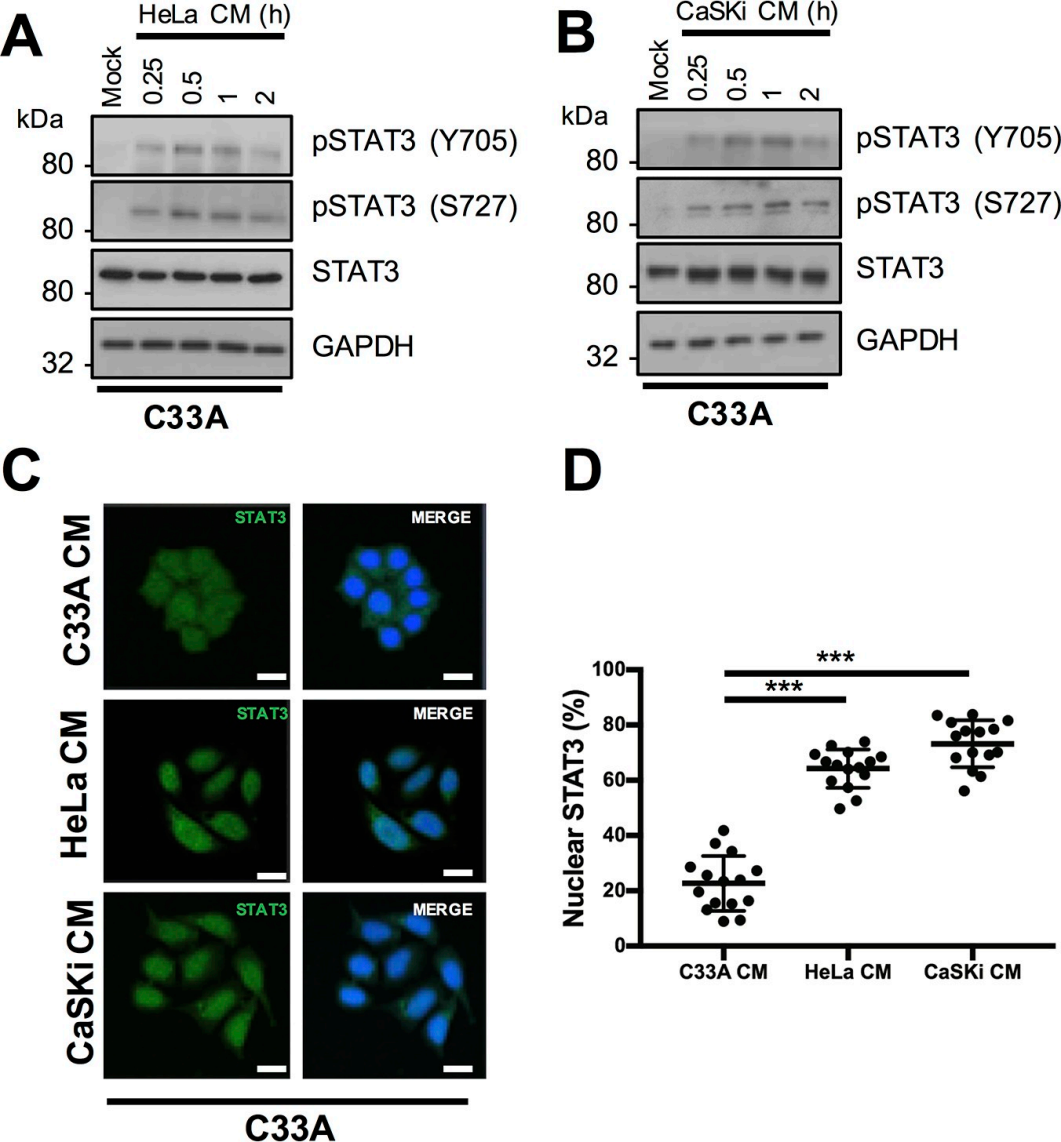
A secreted factor from HPV+ cervical cancer cells can induce STAT3 phosphorylation in HPV- cervical cancer cells. C33A cells were serum starved for 24 hours and conditioned media from **A)** HeLa or **B)** CaSKi cells was added for the indicated time points. For the control, C33A conditioned media was added to cells for 2 hours. Western blots show cell lysates analysed for the expression of phosphorylated and total STAT3. GAPDH served as a loading control. Data are representative of at least three biological independent repeats. **C)** C33A cells were serum starved for 24 hours and incubated with conditioned media from HeLa or CaSKi cells for 2 hours. For the control, C33A conditioned media was added to cells for 2 hours. Cells were analysed by immunofluorescence staining for total STAT3 (green) and counterstained with DAPI to highlight the nuclei (blue in the merged panels). Scale bar 20 μm. **D)** Scatter dot plot of percentage nuclear STAT3 from **C)**. Data represents the percentage nuclear localisation of STAT3 from 15 cells from three independent experiments. Nuclear localisation was calculated using ImageJ [92].

As STAT3 can be regulated by extracellular stimuli, we investigated whether HPV promotes the secretion of factors capable of inducing STAT3 phosphorylation. C33A cells (HPV-) incubated with conditioned media (CM) from HeLa (HPV18+) or CaSKi (HPV16+) cells showed an increase in STAT3 phosphorylation on both Y705 and S727 residues over time compared to treatment with CM from C33A cells (Fig 2A and 2B). STAT3 phosphorylation reached a peak between 30 minutes and 1 hour (Fig 2A and 2B). This was accompanied by a significant increase in STAT3 nuclear accumulation within C33A cells treated with HeLa or CaSKi-CM (Fig 2C; quantified in 2D). These data indicate that a factor(s) secreted into the media from HPV+ cells induces STAT3 phosphorylation in target cells.

### IL-6 secretion is increased in HPV positive cervical cancer cells

**Fig 3:**
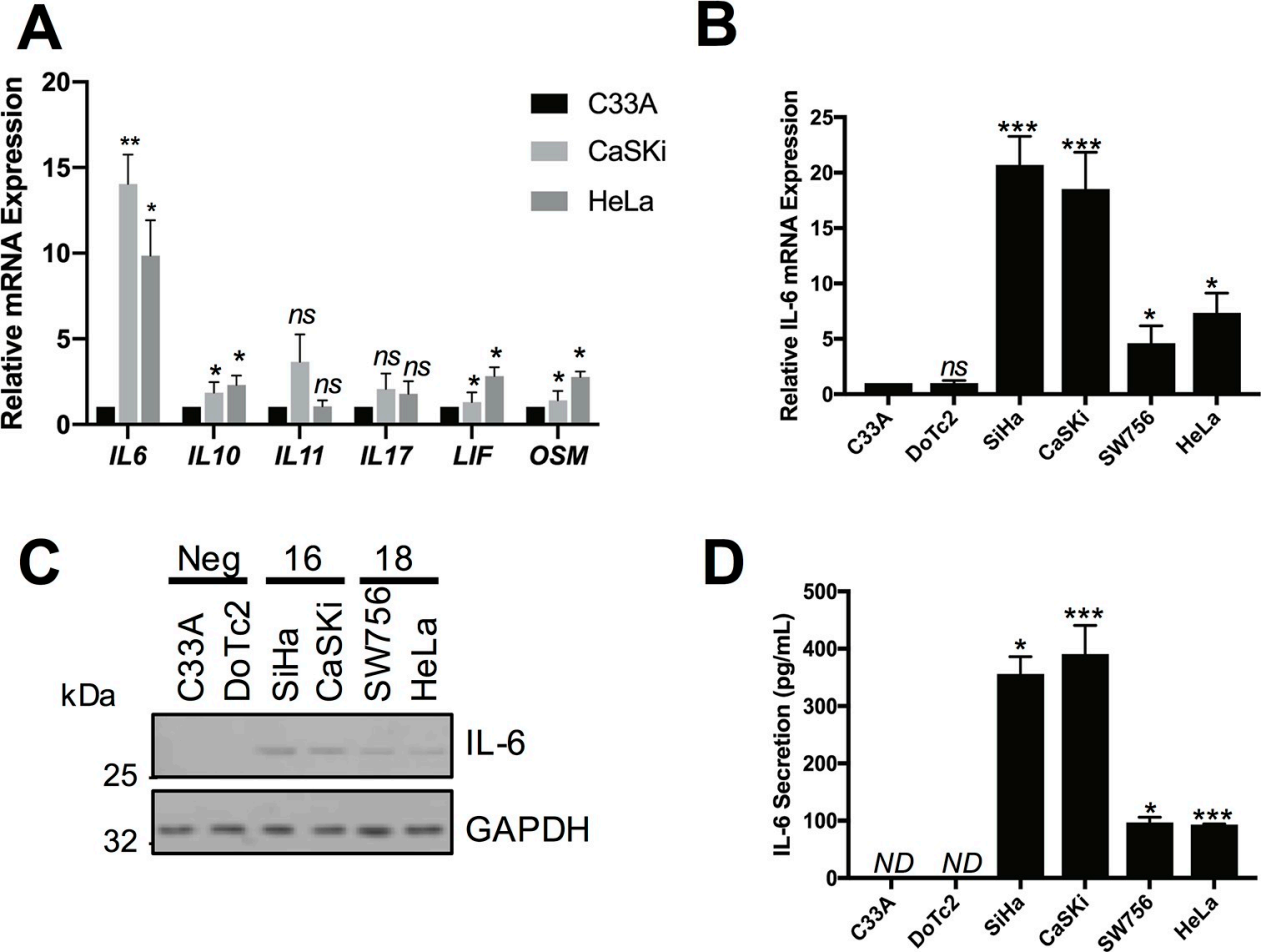
IL-6 is up regulated in HPV+ cervical cancer cells. **A)** Expression levels of cytokines from the IL-6 family were analysed in cervical cell lines by RT-qPCR. Samples were normalized against U6 mRNA levels. Representative data are presented relative to the HPV- cells. Bars are the means ± standard deviation from at least three biological repeats. *P<0.05, **P<0.01 (Student’s t-test). **B)** IL-6 mRNA expression in six cervical cancer cell lines analysed by RT-qPCR. Samples were normalized against U6 mRNA levels. Representative data are presented relative to the HPV- cells. Bars are the means ± standard deviation from at least three biological repeats. *P<0.05, ***P<0.001 (Student’s t-test). **C)** Representative western blot from six cervical cancer cell lines for the expression of IL-6. GAPDH served as a loading control. Data are representative of at least three biological independent repeats. **D)** ELISA analysis from the culture medium from six cervical cancer cell lines for secreted IL-6 protein. Error bars represent the mean ± standard deviation of a minimum of three biological repeats. ND = not determined (below the detection threshold). *P<0.05, ***P<0.001 (Student’s t-test).

To identify the secreted factor responsible for inducing STAT3 phosphorylation, we focused on members of the IL-6 family of pro-inflammatory cytokines, as these have a well-studied role in the activation of STAT3 [27]. Firstly, the mRNA expression levels of key members of the family were measured by RT-qPCR. In both HeLa and CaSKi cells, *IL6*, *IL10*, *LIF* (Leukaemia inhibitory factor) and *OSM* (Oncostatin M) mRNA levels were significantly higher than in C33A cells (Fig 3A), with *IL*6 showing the greatest increase. Building on this, we analysed *IL6* mRNA expression in all six cervical cancer cell lines. HPV16+ and HPV18+ cells displayed a significantly higher level of *IL6* mRNA expression compared with HPV negative cells (Fig 3B), which correlated with intracellular IL-6 protein expression analysed by western blot (Fig 3C). Finally, an IL-6 specific ELISA confirmed that HPV positive but not HPV negative cells secreted IL-6 (Fig 3D). These data indicate that HPV positive cell lines express and secrete significantly higher levels of IL-6 compared with HPV negative cell lines.

### IL-6 binding to gp130-containing receptor complexes is required for STAT3 phosphorylation and nuclear translocation in cervical cancer cells

**Fig 4:**
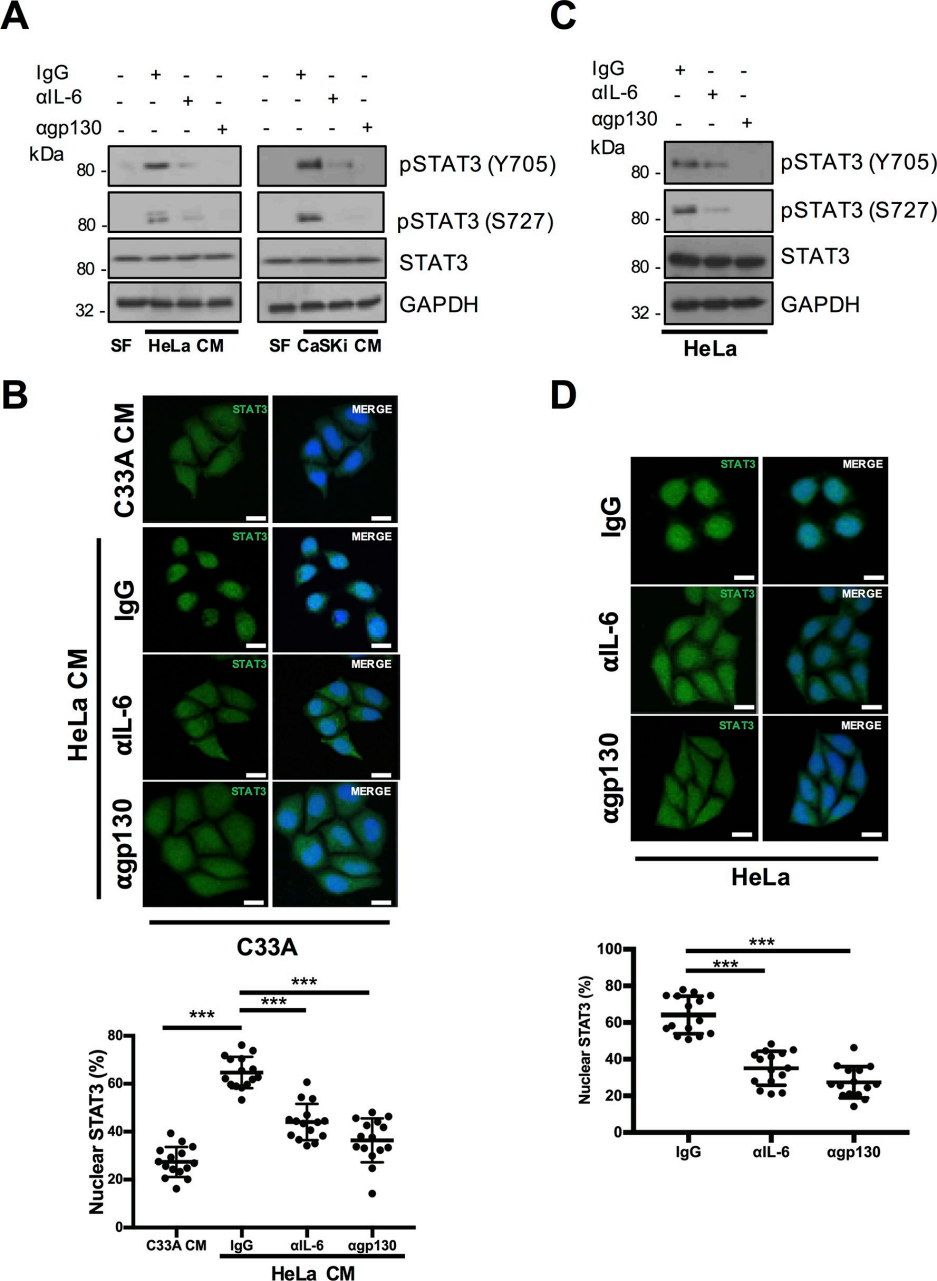
Autocrine/paracrine IL-6 signalling induces STAT3 phosphorylation and nuclear translocation in cervical cancer cell lines. **A)** Representative western blot of C33A cells treated with conditioned medium (CM) from HeLa and CaSKi cells for 2 hours. Cells were pre-treated with IgG, anti-IL6 or anti-gp130 antibody for 4 hours before CM addition. Cell lysates were analysed for phosphorylated and total STAT3 expression. GAPDH served as a loading control. Data are representative of at least three biological independent repeats. **B)** C33A cells treated with conditioned medium (CM) from HeLa and CaSKi cells for 2 hours. Cells were pre-treated with IgG, anti-IL6 or anti-gp130 antibody for 4 hours before CM addition. Cells were then analysed by immunofluorescence staining for total STAT3 (green) and counterstained with DAPI to highlight the nuclei (blue in the merged panels). Scale bar 20 μm. Quantification of nuclear STAT3 is shown below. **C)** HeLa cells were treated with IgG, anti-IL6 or anti-gp130 for 4 hours. Cell lysates were analysed for phosphorylated and total STAT3 expression. GAPDH served as a loading control. Data are representative of at least three biological independent repeats. **D)** HeLa cells were treated with IgG, anti-IL6 or anti-gp130 for 4 hours. Cells were then analysed by immunofluorescence staining for total STAT3 (green) and counterstained with DAPI to highlight the nuclei (blue in the merged panels). Scale bar 20 μm. Quantification of nuclear STAT3 is shown below.

IL-6 signalling is initiated by an interaction between IL-6 and the IL-6 receptor (IL-6R)–gp130 co-receptor complex [21]. IL-6 and gp130 blocking antibodies were utilised to interrogate their requirement for STAT3 phosphorylation in cervical cancer cells. Firstly, we confirmed that IL-6 mediated STAT3 phosphorylation in C33A cells treated with HPV positive CM by pre-incubating with the gp130 blocking antibody before treatment. Separately, the IL-6 neutralising antibody was added to CM before addition to C33A cells. Both treatments reduced HPV positive CM induced STAT3 phosphorylation (Fig 4A) and nuclear translocation (Fig 4B). Thus, IL-6 secreted from HPV positive cervical cancer cells can induce the activation of STAT3 in HPV negative cervical cancer cells via IL-6/gp130 signalling.

To confirm that autocrine IL-6/gp130 signalling was also required for STAT3 activation in HPV+ lines, HeLa cells were pre-incubated with IL-6 and gp130 neutralizing antibodies. Incubation with either neutralising antibody reduced STAT3 dual phosphorylation and nuclear translocation (Fig 4C and 4D), confirming an autocrine mechanism of STAT3 activation. Together, these data demonstrate that HPV-induced IL-6 causes autocrine and paracrine activation of STAT3 signalling in cervical cell lines.

### HPV E6 mediated induction of IL-6 expression is necessary and sufficient for STAT3 phosphorylation

**Fig 5:**
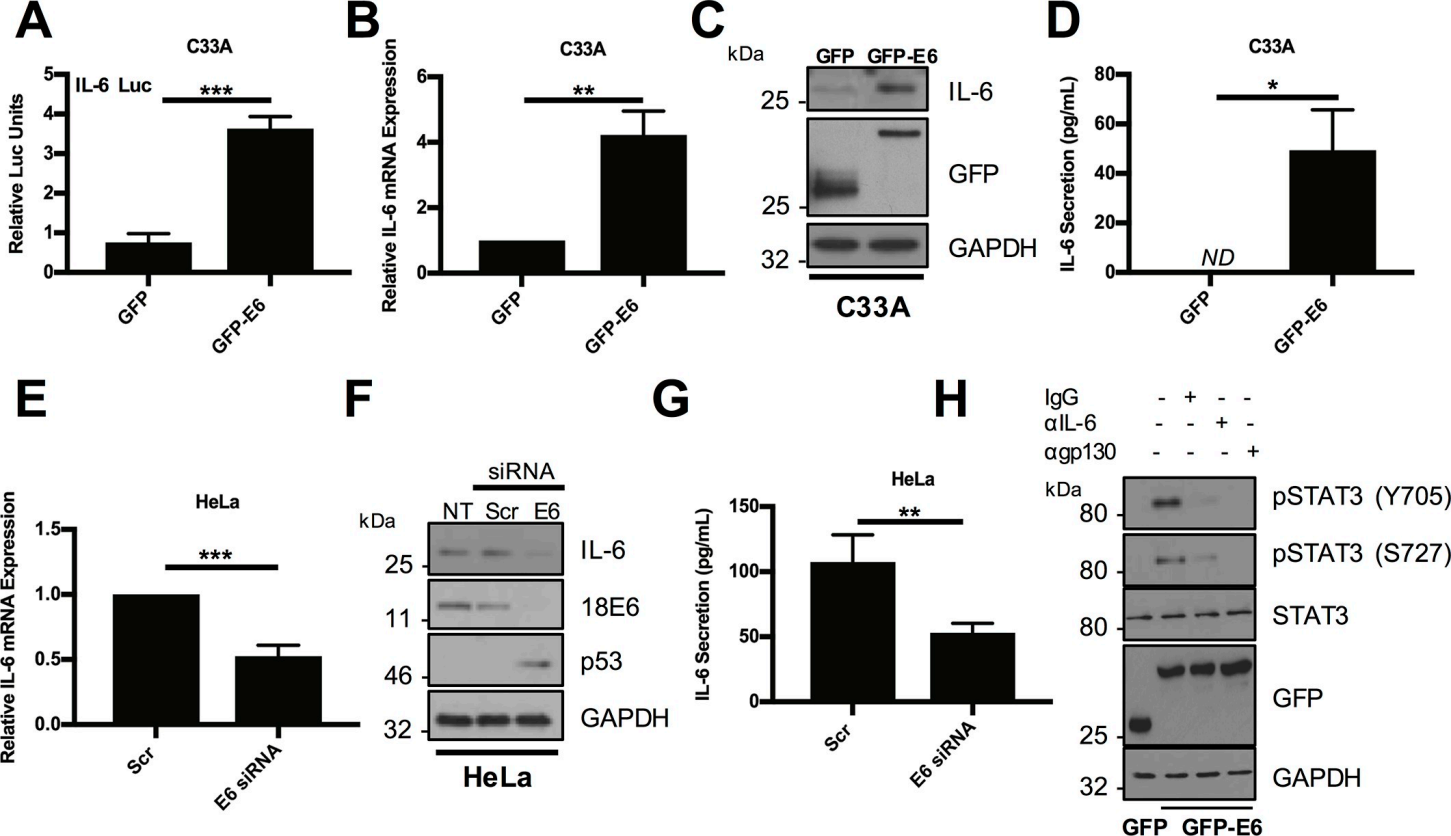
HPV18 E6 induced IL-6 expression is required for STAT3 phosphorylation. **A)** Representative luciferase reporter assay from C33A cells co-transfected with GFP tagged HPV18 E6 and an IL-6 promoter reporter. Promoter activity was measured using a dual-luciferase system. Data are presented as relative to the GFP transfected control. **B)** C33A cells were transiently transfected with GFP or GFP tagged HPV18 E6 and RNA was extracted for RT-qPCR analysis of IL-6 expression. Samples were normalized against U6 mRNA levels. Data are presented relative to the GFP control. **C)** Representative western blot of C33A cells transiently transfected with GFP or GFP tagged HPV18 E6 and analysed for IL-6 expression. Expression of HPV E6 was confirmed using a GFP antibody and GAPDH served as a loading control. **D)** C33A cells were transiently transfected with GFP or GFP tagged HPV18 E6. The culture medium was analysed for IL-6 protein by ELISA. **E)** HeLa cells were transfected with a pool of two specific siRNAs targeting HPV18 E6 and analysed for IL-6 mRNA expression by RT-qPCR. Samples were normalized against U6 mRNA levels. **F)** Representative western blot of HeLa cells transfected with a pool of two specific siRNAs targeting HPV18 E6 and analysed for the expression of IL-6. Knockdown of HPV18 E6 was confirmed using an antibody against HPV18 E6 and p53. GAPDH served as a loading control. **G)** HeLa cells were transfected with a pool of two specific siRNAs against HPV18 E6. The culture medium was analysed for IL-6 protein by ELISA. **H)** Representative western blot of C33A cells transiently transfected with GFP or GFP tagged HPV18 E6 and treated with IgG, anti-IL6 or anti-gp130 for 4 hours before harvest. Cell lysates were then analysed for phosphorylated and total STAT3. Expression of HPV E6 was confirmed using a GFP antibody and GAPDH served as a loading control. Bars represent the means ± standard deviation from at least three independent biological repeats. *P<0.05, **P<0.01, ***P<0.001 (Student’s t-test).

The increased STAT3 phosphorylation observed in HPV containing normal keratinocytes is driven by the E6 oncoprotein [23]. Additionally, HPV16 E6 has previously been demonstrated to induce IL-6 secretion in non-small cell lung cancer (NSCLC) cells [28]. Therefore, the ability of E6 to induce IL-6 expression in cervical cells was assessed by transfection of C33A cells with an IL-6 promoter luciferase reporter combined with either GFP-E6 or GFP expression plasmids. Expression of HPV18 E6 significantly increased IL-6 promoter activity compared with the GFP control (Fig 5A). This correlated with an increase in endogenous *IL6* mRNA expression (Fig 5B) and IL-6 protein expression (Fig 5C). E6 expression also resulted in a significant increase in IL-6 secretion into the culture media (Fig 5D). To rule out the possibility of the transformed nature of C33A cells contributing to the E6-dependent increase in IL-6 levels, we also expressed HPV18 E6 in primary normal human keratinocytes (NHK). In untransformed cells, E6 increased *IL6* mRNA and protein expression compared to control, and this correlated with an increase in the abundance of IL-6 protein detected in the culture media (S1A–S1C Fig). To demonstrate that endogenous E6 could induce IL-6 expression in HPV+ cancer cells, HeLa (Fig 5) or CaSKi (S2 Fig) cells were treated with two E6 specific siRNAs. Knockdown of E6 led to a significant reduction in *IL6* mRNA expression (Fig 5E and S2A Fig), IL-6 protein expression (Fig 5F and S2B Fig) and secretion (Fig 5G and S2C Fig). To confirm that the E6-mediated activation of STAT3 by IL-6 was also working through the canonical gp130 –IL6R receptor complex, we incubated E6 expressing cells with neutralizing antibodies against gp130 and IL-6. As expected, incubation with either antibody led to a reduction in the E6-mediated phosphorylation of STAT3 (Fig 5H). Activation of STAT3 by HPV18 E6 is independent of interactions with E6AP, p53 and cellular PDZ domains [23]. To address whether IL-6 production requires these functions, wildtype and mutant HPV18 E6 proteins deficient in their ability to bind p53, E6AP or PDZ domains were expressed in C33A cells. As predicted, the mutant E6 proteins increased IL-6 protein expression to levels similar as wildtype (S3 Fig). Together, these data demonstrate that IL-6 expression and secretion are increased by a mechanism independent of the p53 destabilising, and PDZ binding functions, of E6.

### HR-HPV E6 activates NFκB to induce IL-6 expression

**Fig 6:**
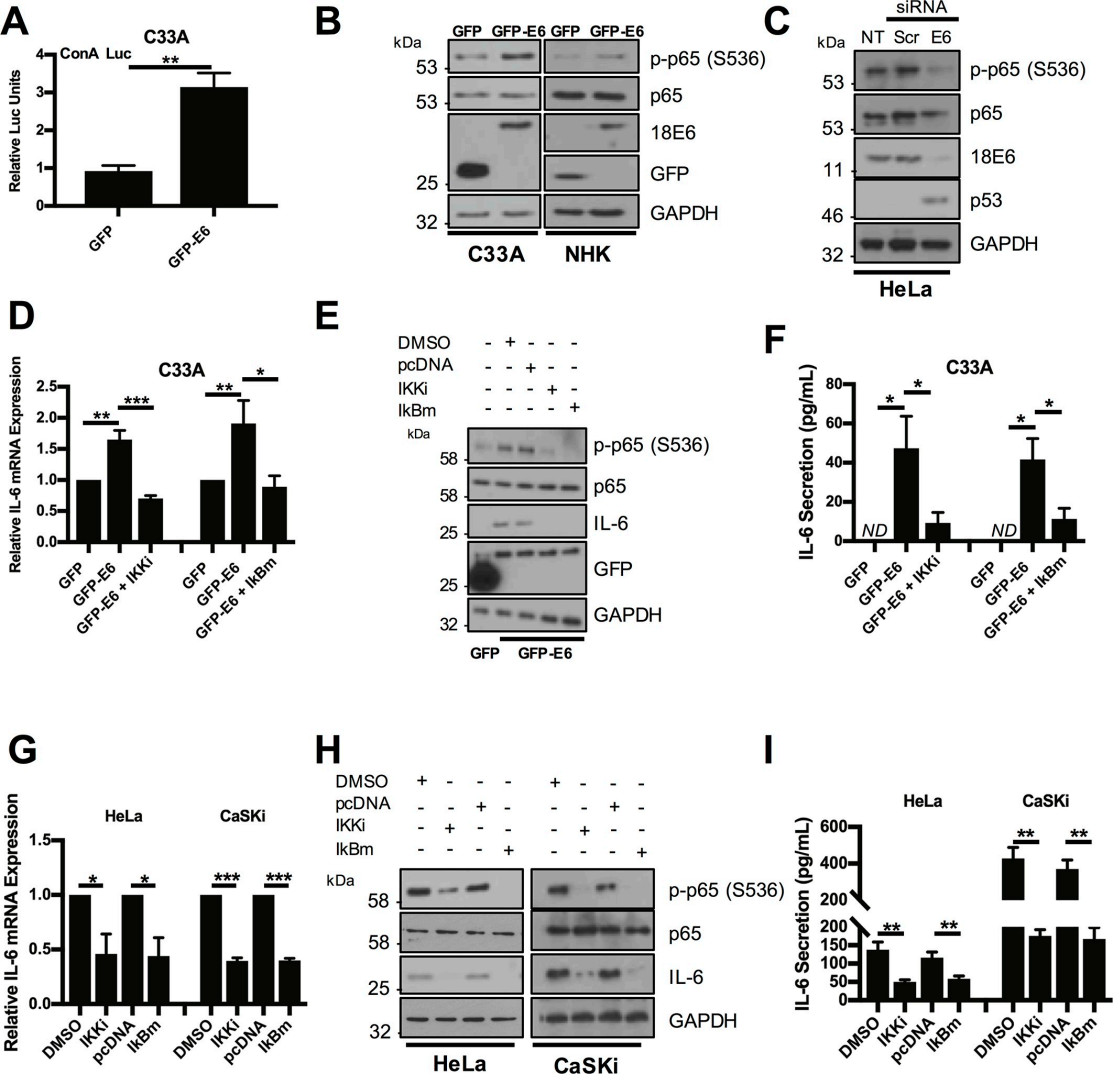
HPV E6 mediated IL-6 expression requires active NFκB. **A)** Representative luciferase reporter assay from C33A cells co-transfected with GFP tagged E6 and a ConA reporter containing tandem κB binding sites [93]. Promoter activity was measured using a dual-luciferase system. Data are presented as relative to the GFP transfected control. **B)** Representative western blot of C33A and normal human keratinocytes (NHK) cells transiently transfected with GFP or GFP tagged HPV18 E6 and analysed for phosphorylated and total p65 expression. Expression of HPV E6 was confirmed using a GFP antibody in the C33A cells. In NHK cells an antibody directly detecting HPV18 E6 was used to confirm expression of the GFP E6 fusion protein. GAPDH served as a loading control. **C)** Representative western blot of HeLa cells transfected with a pool of two specific siRNAs targeting HPV18 E6 and analysed for the expression of phosphorylated and total p65. Knockdown of HPV18 E6 was confirmed using an antibody against HPV18 E6 and p53. GAPDH served as a loading control. **D-F)** C33A cells were co-transfected with GFP, GFP tagged HPV18 E6 or GFP tagged HPV18 E6 and mutant IκBα (IκBm). Cells were then either left untreated or treated with IKK inhibitor VII (IKKi). **D)** Total RNA was extracted for RT-qPCR analysis of IL-6 expression. Samples were normalized against U6 mRNA levels. Representative data are presented relative to the GFP control. **E)** Cell lysates were analysed for the expression of phosphorylated and total p65 and IL-6. Expression of HPV E6 was confirmed using a GFP antibody and GAPDH served as a loading control. **F)** The culture medium was analysed for IL-6 protein by ELISA. **G-I)** HeLa and CaSKi cells were transfected with mutant IκBα (IκBm) or treated with IKK inhibitor VII (IKKi). **G)** Total RNA was extracted for RT-qPCR analysis of IL-6 expression. Samples were normalized against U6 mRNA levels. Representative data are presented relative to the DMSO or pcDNA control. **H)** Cell lysates were analysed for the expression of phosphorylated and total p65 and IL-6. GAPDH served as a loading control. **I)** The culture medium was analysed for IL-6 protein by ELISA. Bars represent the means ± standard deviation from at least three independent biological repeats. *P<0.05, **P<0.01, ***P<0.001 (Student’s t-test).

The NFκB transcription factor is an important regulator of IL-6 expression, which is activated in response to a range of extracellular ligands such as TNFα [29]. HR-E6 has previously been shown to activate NFκB signalling under hypoxic conditions [30,31]. However, to assess whether NFκB is necessary for increased IL-6 expression under normoxic conditions, we first tested whether expression of HPV18 E6 in isolation would activate NFκB in C33A cells. Using an NFκB driven luciferase reporter plasmid, overexpression of E6 induced NFκB activity compared to a GFP control (Fig 6A). Canonical NFκB signalling results in the phosphorylation of the p65 subunit and its nuclear translocation, where it is transcriptionally active in complex with additional NFκB subunits including p50 [29]. E6 over-expression in C33A or NHK cells induced robust p65 phosphorylation, without affecting total p65 protein levels (Fig 6B). In contrast, siRNA knockdown of E6 in HeLa cells reduced p65 phosphorylation (Fig 6C), together suggesting that HPV E6 activates canonical NFκB.

To understand the role of NFκB in E6-driven IL-6 production, we employed a dual approach to prevent NFκB activation in C33A cells overexpressing HPV18 E6. Cells were treated either with a small molecule inhibitor (IKKi) targeting the IKKα/β complex, which phosphorylates and activates NFκB, or transfected with a plasmid encoding a mutant IκBα protein (IκBm), which cannot be degraded and as such retains inactive NFκB in the cytosol [32]. Inhibition of NFκB using either IKKi or IκBm led to a significant reduction in E6-mediated *IL6* mRNA expression (Fig 6D), IL-6 protein levels (Fig 6E) and secretion (Fig 6F). Importantly, both strategies effectively inhibited NFκB activity as judged by a reduction in p65 phosphorylation (Fig 6E).

We also tested if NFκB activity was required for mediating the increased IL-6 levels seen in HeLa and CaSKi cells. In these cells, inhibition of NFκB led to a reduction in *IL6* mRNA expression, IL-6 protein levels and secretion (Fig 6G–6I). Collectively, these data demonstrate that HR-E6-mediated IL-6 expression requires active NFκB.

### NFκB is required for STAT3 phosphorylation in HR-E6 expressing cells

It was necessary to test whether NFκB was also needed for the activation of STAT3. As proof of principle, we tested the ability of the NFκB activator TNFα to induce STAT3 phosphorylation. As expected, treatment of serum starved C33A cells with TNFα caused robust p65 phosphorylation, which peaked at 0.5 hours after treatment (S4 Fig). This was coupled with an increase in IL-6 expression, which remained high up to 24 hours post treatment. Importantly, TNFα treatment also caused an increase in STAT3 phosphorylation and nuclear translocation, observed to peak approximately 2 hours post treatment (S4A and S4B Fig).

**Fig 7:**
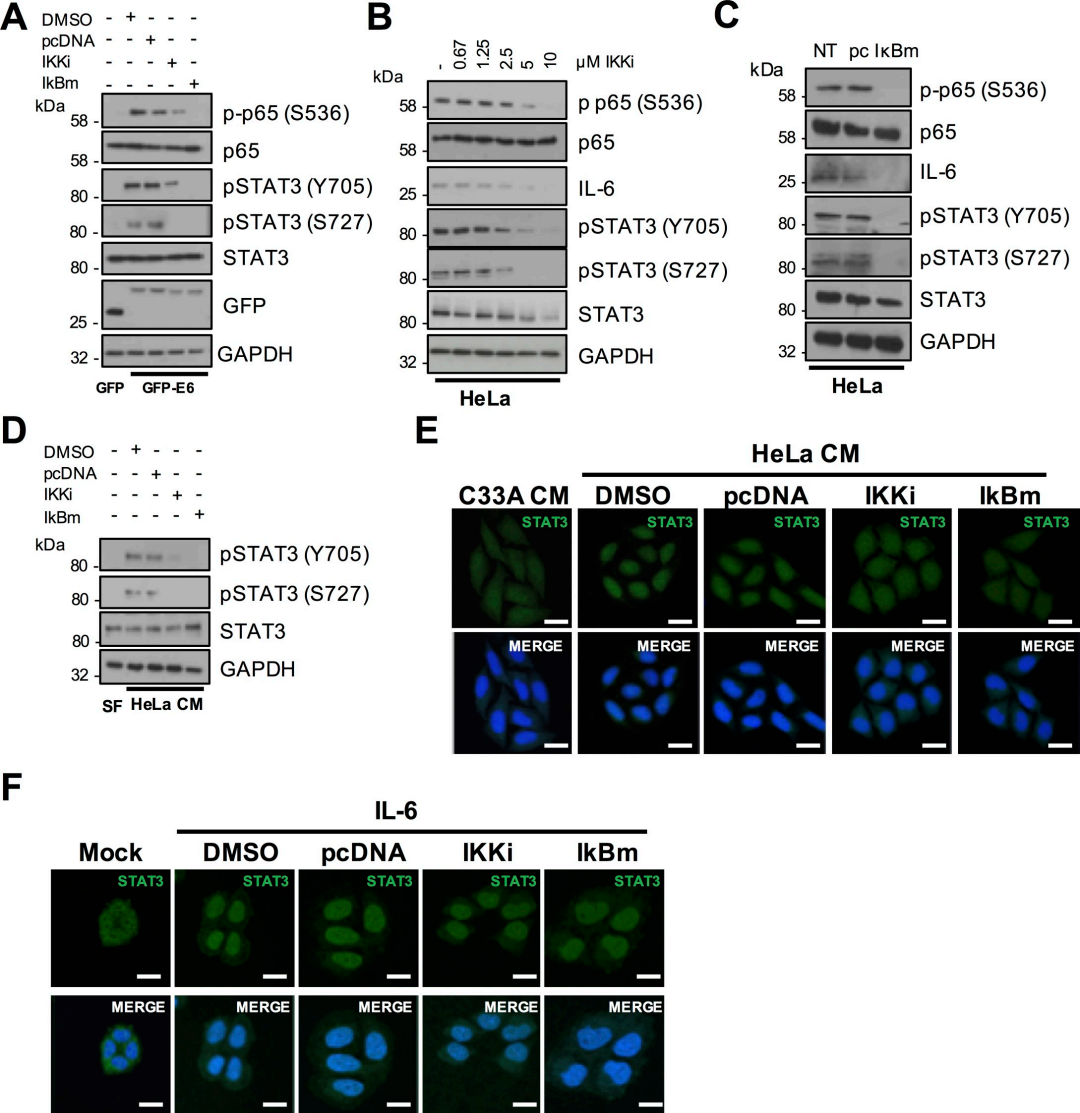
Active NFκB is required for HPV E6 mediated STAT3 signalling. **A)** C33A cells were co-transfected with GFP, GFP tagged HPV18 E6 or GFP tagged HPV18 E6 and mutant IκBα (IκBm). Cells were then either left untreated or treated with IKK inhibitor VII (IKKi). Cell lysates were analysed for phosphorylated and total p65, phosphorylated and total STAT3 expression. Expression of HPV E6 was confirmed using a GFP antibody and GAPDH served as a loading control. **B)** Representative western blot of HeLa cells treated with increasing doses of the IKKα/β inhibitor IKK inhibitor VII (IKKi). Cell lysates were analysed for the expression of phosphorylated and total p65, phosphorylated and total STAT3 and IL-6 expression. GAPDH served as a loading control. **C)** Representative western blot of HeLa cells transfected with mutant IκBα (IκBm). Cell lysates were analysed as in **B). D)** C33A cells were serum starved for 24 hours. Cells were then treated with HeLa conditioned media for 2 hours from HeLa cells treated with DMSO or IKKi or transfected with pcDNA or IκBm. Cell lysates were analysed for phosphorylated and total STAT3 expression. GAPDH served as a loading control. **E)** C33A cells were serum starved for 24 hours. Cells were then treated with HeLa condition media for 2 hours from HeLa cells treated with DMSO or IKKi or transfected with pcDNA or IκBm. Cells were analysed by immunofluorescence staining for total STAT3 (green) and counterstained with DAPI to highlight the nuclei (blue in the merged panels). Scale bar 20 μm. **F)** C33A were treated with DMSO or IKKi or transfected with pcDNA or IκBm before treatment with 20 ng/mL recombinant human IL-6 for 30 mins. Cells were analysed by immunofluorescence staining for total STAT3 (green) and counterstained with DAPI to highlight the nuclei (blue in the merged panels). Scale bar 20 μm.

We next set out to link NFκB activation by E6 to STAT3 phosphorylation. For this, HPV18 E6 was overexpressed in C33A cells, with or without treatment with the NFκB inhibitor IKKi, or co-expression of IκBm. E6 noticeably increased the levels of p65 and STAT3 phosphorylation and inhibition of NFκB by either approach reduced STAT3 phosphorylation (Fig 7A). Blockade of NFκB activity also reduced STAT3 phosphorylation in HeLa (Fig 7B and 7C) and CaSKi (S5A and S5B Fig) cells, suggesting that E6 mediated STAT3 phosphorylation is depended on NFκB activity. To ascertain if NFκB was essential for the paracrine activation of STAT3 in C33A cells, we took CM from HeLa cells in which NFκB was inhibited and added this to C33A cells. This failed to induce STAT3 phosphorylation (Fig 7D) and nuclear translocation (Fig 7E; quantified in S6A Fig). Importantly, inhibition of NFκB activity had no effect on STAT3 nuclear translocation mediated by treatment with exogenous IL-6 (Fig 7F; quantified in S6B Fig), demonstrating that NFκB is upstream of IL-6 secretion. Together, these data suggest that NFκB is required for the autocrine and paracrine activation of STAT3.

### The protein kinase AKT is activated by HR-E6 and contributes to NFκB activation and IL-6 production

**Fig 8:**
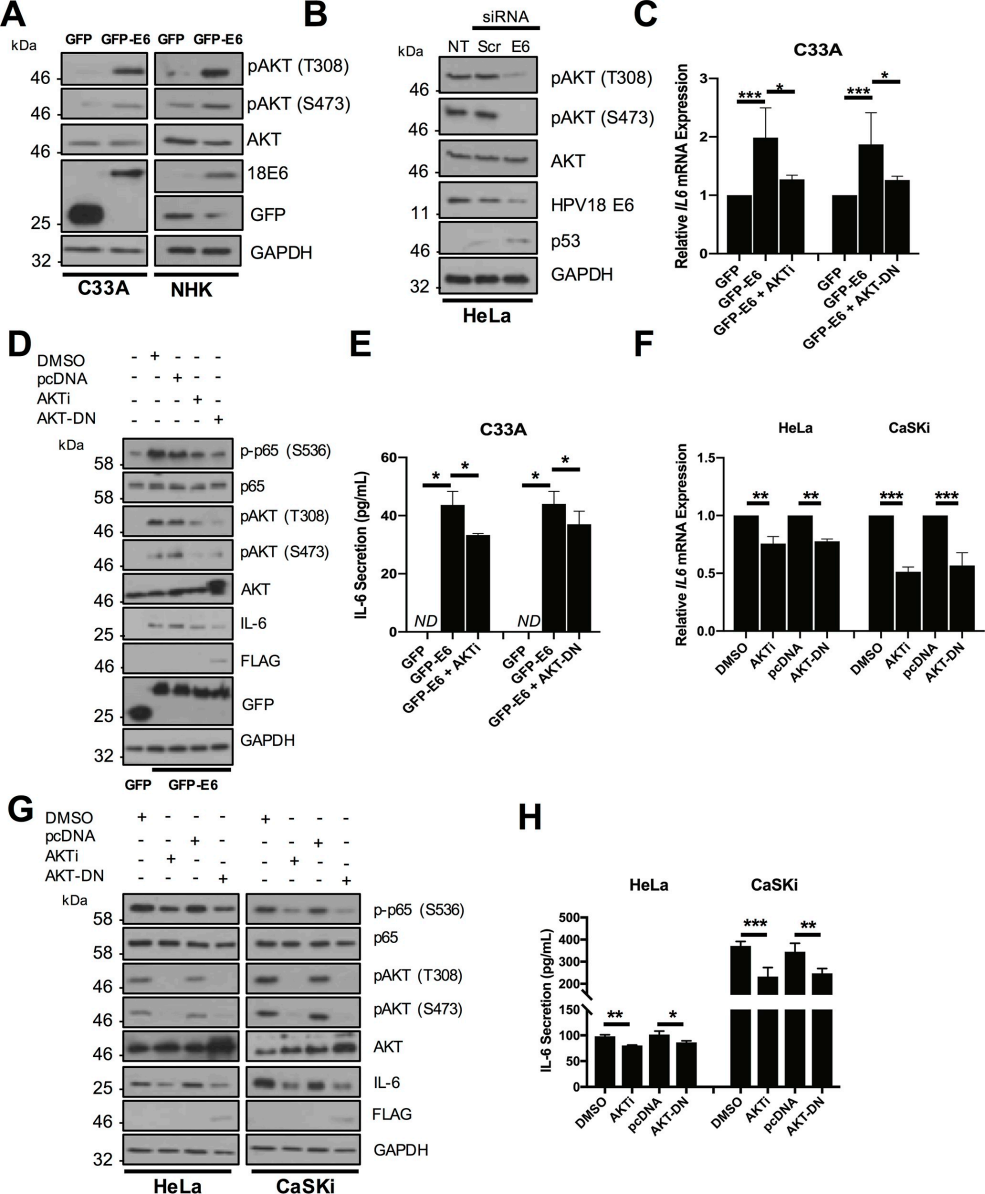
Activation of AKT by HPV E6 contributes to IL-6 expression via NFκB. **A)** Representative western blot of C33A and NHK cells transiently transfected with GFP or GFP tagged HPV18 E6 and analysed for phosphorylated and total AKT expression. Expression of HPV E6 was confirmed in C33A cells using a GFP antibody and in NHK cells using a HPV18 E6 antibody. GAPDH served as a loading control. **B)** Representative western blot of HeLa cells transfected with a pool of two specific siRNAs against HPV18 E6 and analysed for the expression of phosphorylated and total AKT. Knockdown of HPV18 E6 was confirmed using an antibody against HPV18 E6 and p53. GAPDH served as a loading control. **C-E)** C33A cells were co-transfected with GFP, GFP tagged HPV18 E6 or GFP tagged HPV18 E6 and dominant negative AKT (AKT-DN). Cells were either left untreated or treated with AKT inhibitor VIII (AKTi). **C)** Total RNA was extracted for RT-qPCR analysis of IL-6 expression. Samples were normalized against U6 mRNA levels. Representative data are presented relative to the GFP control. **D)** Cell lysates were analysed for the expression of phosphorylated and total p65 and AKT and IL-6 protein. Expression of HPV E6 was confirmed using a GFP antibody, the AKT-DN was detected using an antibody against the FLAG epitope and GAPDH served as a loading control. **E)** The culture medium was analysed for IL-6 protein by ELISA **F-H)** HeLa and CaSKi cells transfected with AKT-DN or treated with AKTi. **F)** Total RNA was extracted for RT-qPCR analysis of IL-6 expression. Samples were normalized against U6 mRNA levels. Representative data are presented relative to the GFP control. **G)** Cell lysates were analysed for the expression of phosphorylated and total p65 and AKT and IL-6 protein. Expression of HPV E6 was confirmed using a GFP antibody and AKT-DN using the FLAG epitope. GAPDH served as a loading control. **H)** The culture medium was analysed for IL-6 protein by ELISA. Bars represent the means ± standard deviation from at least three independent biological repeats. *P<0.05, **P<0.01, ***P<0.001 (Student’s t-test).

NFκB is activated by multiple upstream signalling components in a stimulus and tissue-dependent manner [33]. In searching for upstream activators, we initially focused on known targets of HR-E6 with a link to NFκB signalling. The PI3K/AKT signalling pathway is frequently activated in cervical cancers due to mutations in the *PIK3CA* gene [34], and AKT can activate NFκB and mediate IL-6 expression in some cancers [35–37]. Finally, AKT has previously been shown to be activated by E6 [38]. We therefore tested whether AKT was involved in NFκB activation and IL-6 secretion in HPV positive cervical cancer cell lines. First, we confirmed that E6 activates AKT, as measured by an increase in AKT phosphorylation. Over expression of HPV18 E6 in C33A and NHK cells led to a marked increase in AKT phosphorylation at both threonine 308 and serine 473, without affecting levels of total AKT protein (Fig 8A). Conversely, siRNA knockdown of E6 in HeLa cells reduced AKT phosphorylation (Fig 8B), confirming that HPV18 E6 activates AKT.

To interrogate the contribution of AKT activation to IL-6 production, E6 expressing cells were treated either with a potent allosteric inhibitor of AKT (AKTi), targeting the AKT1 and 2 isoforms [39] or transfected with a plasmid encoding a catalytically inactive AKT mutant (AKT-DN) [40]. Inhibition of AKT by either approach led to a statistically significant reduction in *IL6* mRNA expression (Fig 8C), coupled to a smaller loss in protein expression and secretion (Fig 8D and 8E). To confirm that the AKT-mediated increase in IL-6 was transduced via NFκB, we measured p65 phosphorylation levels in E6 expressing C33A cells treated with either AKTi or co-transfected with AKT-DN. As expected, a loss of AKT phosphorylation was observed, indicating that the inhibition strategy was successful, and this was coupled with a reduction in IL-6 protein expression (Fig 8D) and a partial reduction in p65 phosphorylation, suggesting that AKT lies upstream of NFκB in the regulation of IL-6.

We also validated the impact of AKT inhibition on IL-6 production in both HeLa and CaSKi cells. Interestingly, inhibition of AKT in CaSKi cells had a greater effect in reducing *IL6* mRNA expression (Fig 8F), IL-6 protein levels (Fig 8G) and secretion (Fig 8H) than in HeLa cells. Furthermore, AKT inhibition led to a greater reduction in p65 phosphorylation in CaSKi cells (Fig 8G). These data confirm that AKT contributes to IL-6 production through the regulation of NFκB. Strikingly, there appear to be cell line differences in the requirement for AKT, with ablation of AKT kinase activity having a more significant impact on signalling in CaSKi than in HeLa cells.

### AKT contributes to STAT3 phosphorylation in cells expressing E6 and in HPV positive cervical cell lines

**Fig 9:**
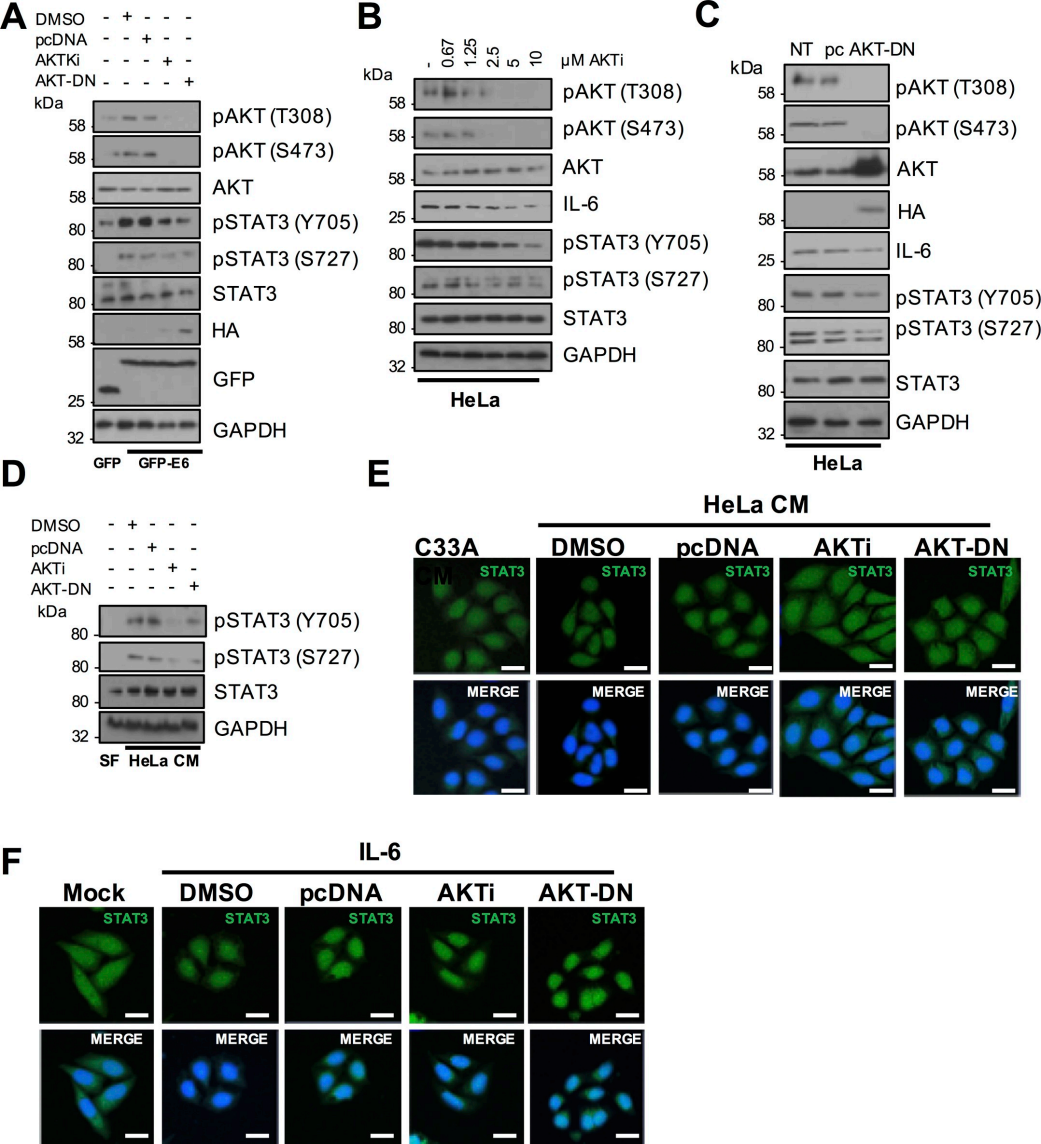
Active AKT contributes to HPV E6 mediated STAT3 signalling. **A)** C33A cells were co-transfected with GFP, GFP tagged HPV18 E6 or GFP tagged HPV18 E6 and dominant negative AKT (AKT-DN). Cells were then either left untreated or treated with AKT inhibitor VIII (AKTi). Cell lysates were analysed for phosphorylated and total AKT, and phosphorylated and total STAT3 expression. Expression of HPV E6 was confirmed using a GFP antibody and GAPDH served as a loading control. **B)** Representative western blot of HeLa cells treated with increasing doses of the AKTi. Cell lysates were analysed for the expression of phosphorylated and total AKT, phosphorylated and total STAT3 and IL-6 expression. GAPDH served as a loading control. **C)** Representative western blot of HeLa cells transfected with dominant negative AKT (AKT-DN). Cell lysates were analysed as in **B). D)** C33A cells were serum starved for 24 hours. Cells were then treated with HeLa conditioned media for 2 hours from HeLa cells treated with DMSO or AKTi or transfected with pcDNA or AKT-DN. Cell lysates were analysed for phosphorylated and total STAT3 expression. GAPDH served as a loading control. **E)** C33A cells were serum starved for 24 hours. Cells were then treated with HeLa condition media for 2 hours from HeLa cells treated with DMSO or AKTi or transfected with pcDNA or AKT-DN. Cells were analysed by immunofluorescence staining for total STAT3 (green) and counterstained with DAPI to highlight the nuclei (blue in the merged panels). Scale bar 20 μm. **F)** C33A were treated with DMSO or AKTi or transfected with pcDNA or AKT-DN before treatment with 20 ng/mL recombinant human IL-6 for 30 mins. Cells were analysed by immunofluorescence staining for total STAT3 (green) and counterstained with DAPI to highlight the nuclei (blue in the merged panels). Scale bar 20 μm.

Based on the observation that inhibition of AKT reduced IL-6 production, particularly in CaSKi cells, we tested whether blockade of AKT activity would also impact on STAT3 phosphorylation. HPV18 E6 expressing C33A cells treated with AKTi or co-expressing AKT-DN showed a loss of AKT phosphorylation coupled to a modest reduction in STAT3 phosphorylation at both residues (Fig 9A). Levels of STAT3 phosphorylation were also measured in HeLa and CaSKi cells treated with increasing concentrations of AKTi or over-expressing the AKT-DN protein. Whilst these inhibitory strategies reduced AKT phosphorylation in both cell lines, the corresponding reduction in STAT3 phosphorylation in HeLa cells was minor and mirrored the small reduction in IL-6 protein expression (Fig 9B and 9C). In contrast, loss of AKT activity corresponded with a greater reduction in IL-6 expression and STAT3 phosphorylation in CaSKi cells (S7A and S7B Fig). To ascertain if AKT was essential for the paracrine activation of STAT3 in C33A cells, we took CM from HeLa cells in which AKT was inhibited either by treatment with AKTi or co-expression of AKT-DN and added this to C33A cells. CM from cells with inhibited AKT caused less STAT3 phosphorylation (Fig 9D) and nuclear translocation (Fig 9E; quantified in S8A Fig) compared to controls. Importantly, inhibition of AKT activity had no effect on STAT3 nuclear translocation mediated by exogenous IL-6 (Fig 9F; quantified in S8B Fig), demonstrating that AKT is upstream of IL-6 secretion. Together, these data suggest that AKT is an E6 effector playing a minor role in the autocrine and paracrine activation of STAT3.

### Rac1 mediates NFκB activation and IL-6 production in HPV positive cervical cancer cells

**Fig 10:**
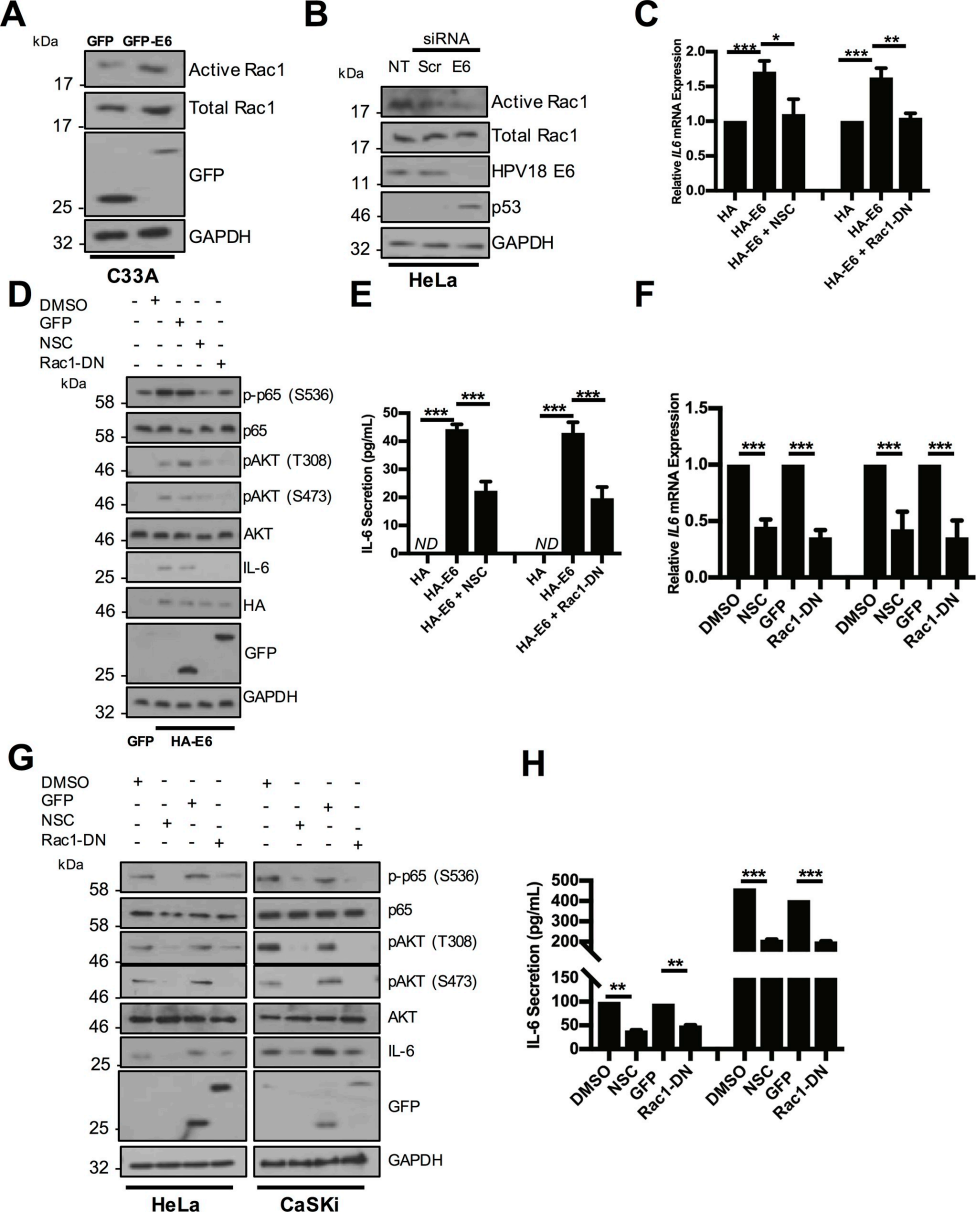
Rac1 is upstream of AKT/NFκB in HPV E6 mediated IL-6 production. **A)** Representative western blot of C33A cells transiently transfected with GFP or GFP tagged HPV18 E6 and analysed for active Rac1 using a Rac1 activity assay. Expression of HPV E6 was confirmed using a GFP antibody. GAPDH served as a loading control. **B)** Representative western blot of HeLa cells transfected with a pool of two siRNAs targeting HPV18 E6 and analysed for active Rac1 using a Rac1 activity assay. Knockdown of HPV18 E6 was confirmed using an antibody against HPV18 E6 and p53. GAPDH served as a loading control. **C-E)** C33A cells were co-transfected with GFP, HA tagged HPV18 E6 alone or co-transfected with Rac1 N17 (Rac1-DN). Cells were then either left untreated or treated with the Rac1 inhibitor NSC23866 (NSC). **C)** Total RNA was extracted for RT-qPCR analysis of IL-6 expression. Samples were normalized against U6 mRNA levels. Representative data are presented relative to the GFP control. **D)** Cell lysates were analysed for the expression of phosphorylated and total p65, phosphorylated and total AKT and IL-6 protein. Expression of HPV E6 was confirmed using a HA antibody, Rac1-DN expression was confirmed using a GFP antibody and GAPDH served as a loading control. **E)** The culture medium was analysed for IL-6 protein by ELISA. **F-H)** HeLa and CaSki cells transfected with Rac1-DN or treated with NSC. **F)** Total RNA was extracted for RT-qPCR analysis of IL-6 expression. Samples were normalized against U6 mRNA levels. Representative data are presented relative to the GFP control. **G)** Cell lysates were analysed for the expression of phosphorylated and total p65, phosphorylated and total AKT and IL-6. Expression of Rac1-DN was confirmed using a GFP antibody and GAPDH served as a loading control. **H)** The culture medium was analysed for IL-6 protein by ELISA. Bars represent the means ± standard deviation from at least three independent biological repeats. *P<0.05, **P<0.01, ***P<0.001 (Student’s t-test).

Whilst our data points to a role for AKT as an E6-effector protein with the potential to activate NFκB and IL-6 production, it is clear that other cellular factors are required and likely play a more prominent role in the signalling pathway. As part of our efforts to identify such candidates, we investigated the possible involvement of the small GTPase Rac1 as it has previously been shown to regulate both AKT and STAT3 signalling [41–44]. In addition, Rac1 is active in a number of skin and oral SCCs, and experiments from mouse models suggest that it acts as a proto-oncogene [45–47]. Rac1 can facilitate papilloma formation in HPV negative 8 transgenic mice [48] and is active in HPV-mediated respiratory papillomatosis [49,50]. We therefore measured the levels of active Rac1 (Rac1-GTP) in E6-expressing C33A cells using an affinity precipitation assay [51]. Expression of HPV18 E6 increased Rac1-GTP levels compared with control cells (Fig 10A) and siRNA depletion of E6 from HeLa cells resulted in a reduction in active Rac1 compared to a scramble control (Fig 10B). Together, these data indicate that E6 activates Rac1.

We next set out to determine if the Rac1 GTPase links E6 to IL-6 production. To this end we used two well characterised approaches to inhibit Rac1 function. C33A cells expressing HPV18 E6 were treated with the Rac1 inhibitor NSC23766 (NSC) or co-transfected with a transdominant mutant Rac1 (Rac1 T17N - Rac1-DN) and IL-6 mRNA levels were measured by RT-qPCR. Inhibition of Rac1 activity with either NSC or Rac1-DN potently inhibited IL-6 mRNA expression in E6 expressing cells (Fig 10C). Inhibition of Rac1 function also impaired E6-induced p65 phosphorylation, supporting a role for Rac1 in the E6 stimulation of NFκB (Fig 10D). Rac1 inhibition was accompanied by a significant reduction in IL-6 protein expression and secretion into the culture media (Fig 10D and 10E). Active Rac1 was also critical for p65 phosphorylation, *IL6* mRNA expression and IL-6 protein expression and secretion in both HeLa and CaSKi cells (Fig 10F–10H). We also noted that blockade of Rac1 function reduced AKT dual phosphorylation either in cells expressing HPV18 E6 in isolation or in HPV positive cervical cancer cells (Fig 10D and 10G). Collectively the results indicate a key role for Rac1 in E6-regulated IL-6 production upstream of AKT and NFκB.

### Rac1 activates STAT3 by an IL-6-dependent mechanism

**Fig 11:**
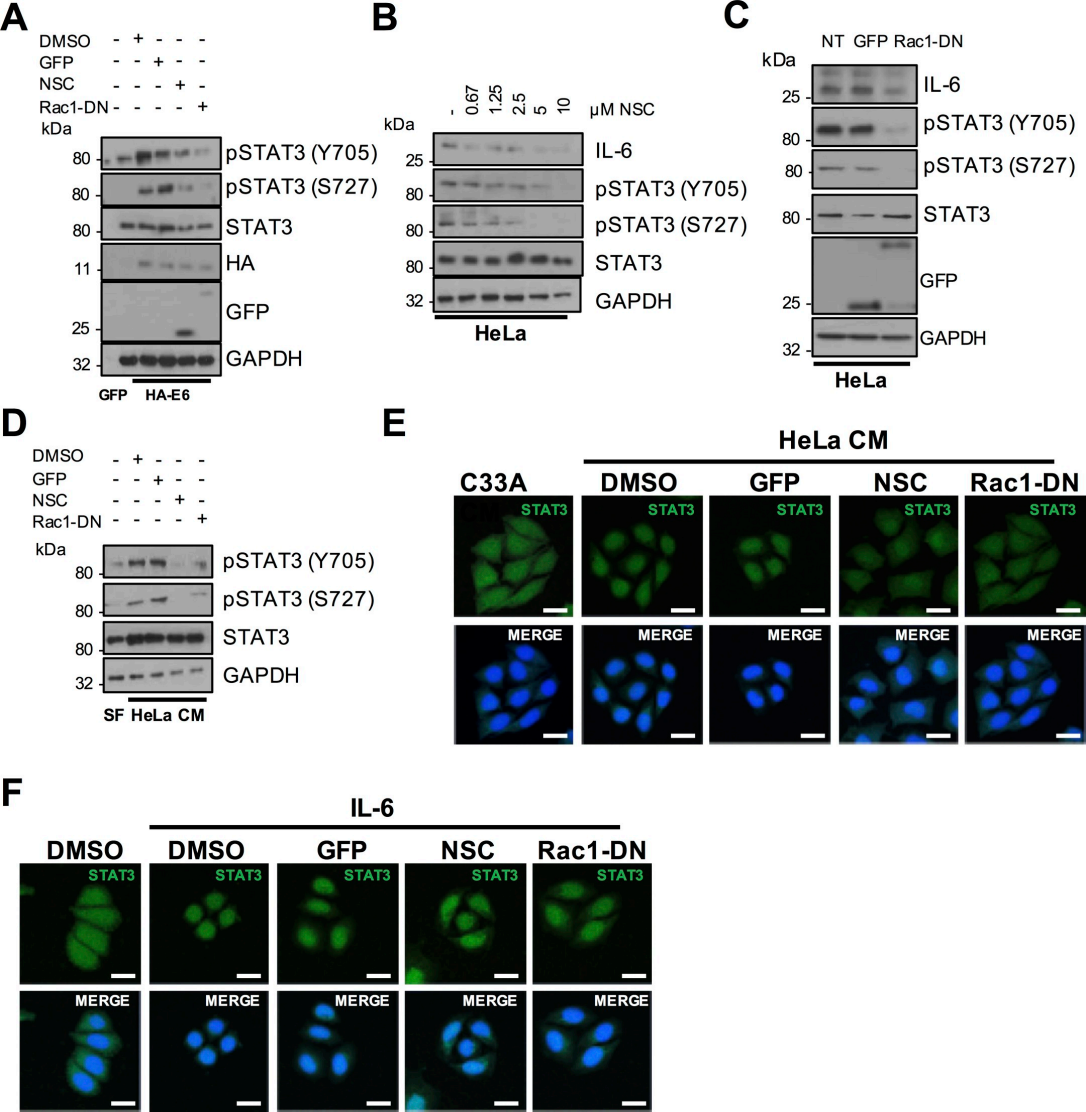
Rac1 is required for HPV E6 mediated IL-6/STAT3 signalling. **A)** C33A cells were co-transfected with GFP, HA tagged HPV18 E6 or HA tagged HPV18 E6 and Rac1-DN. Cells were then either left untreated or treated with the Rac1 inhibitor NSC23866 (NSC). Cell lysates were then analysed for phosphorylated and total STAT3 expression. Expression of HPV E6 was confirmed using a HA antibody, Rac1-DN expression was confirmed using a GFP antibody and GAPDH served as a loading control. **B)** Representative western blot of HeLa cells treated with increasing doses of the NSC. Cell lysates were analysed for the expression of phosphorylated and total STAT3 and IL-6 expression. GAPDH served as a loading control. **C)** Representative western blot of HeLa cells transfected with Rac1-DN. Cell lysates were analysed as in **B). D)** C33A cells were serum starved for 24 hours. Cells were then treated with HeLa conditioned media for 2 hours from HeLa cells treated with DMSO or NSC or transfected with GFP or Rac1-DN. Cell lysates were analysed for phosphorylated and total STAT3 expression. GAPDH served as a loading control. **E)** C33A cells were serum starved for 24 hours. Cells were then treated with HeLa condition media for 2 hours from HeLa cells treated with DMSO or NSC or transfected with GFP or Rac1-DN. Cells were analysed by immunofluorescence staining for total STAT3 (green) and counterstained with DAPI to highlight the nuclei (blue in the merged panels). Scale bar 20 μm. **F)** C33A were treated with DMSO or NSC or transfected with GFP or Rac1-DN before treatment with 20 ng/mL recombinant human IL-6 for 30 mins. Cells were analysed by immunofluorescence staining for total STAT3 (green) and counterstained with DAPI to highlight the nuclei (blue in the merged panels). Scale bar 20 μm.

We examined whether Rac1 was also necessary for STAT3 activation by E6. Chemical inhibition of Rac1 or over-expression of the Rac1-DN mutant decreased the dual phosphorylation of STAT3 in HPV18 E6 expressing C33A cells (Fig 11A). Similarly, NSC addition to HeLa (Fig 11B) or CaSKi (S9A Fig) resulted in a dose-dependent decrease in STAT3 phosphorylation. Confirmatory results were obtained in cells overexpressing the transdominant Rac1 mutant (Fig 11C and S9B Fig). To explore if Rac1 contributed to the paracrine activation of STAT3 in C33A cells, we took CM from HeLa cells in which Rac1 was inhibited and added this to C33A cells. This failed to induce STAT3 phosphorylation (Fig 11D) and nuclear translocation (Fig 11E; quantified in S10A Fig). Crucially, Rac1 inhibition had negligible effect on STAT3 nuclear translocation mediated by treatment with exogenous IL-6 (Fig 11F; quantified in S10B Fig), demonstrating that Rac1 is upstream of IL-6 secretion.

### STAT3 is required for HPV positive cervical cancer cell proliferation

**Fig 12:**
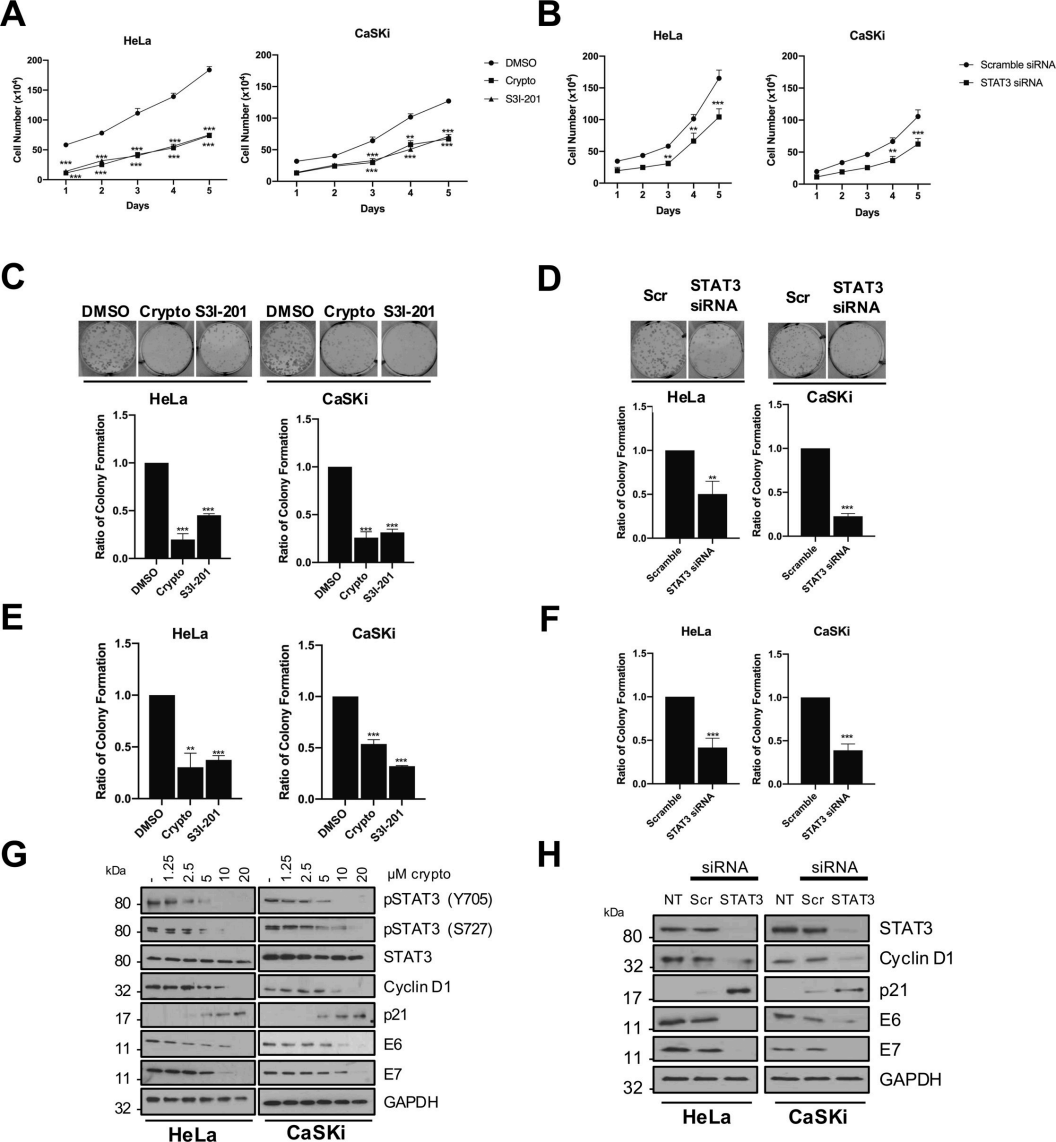
STAT3 is required for the proliferation of HPV+ cervical cancer cells. **A)** Growth curve analysis of HeLa (left) and CaSKi (right) cells after addition of small molecule STAT3 inhibitors for 24 hours. **B)** Growth curve analysis of HeLa (left) and CaSKi (right) cells after transfection of a pool of four specific STAT3 siRNA for 72 hours. **C)** Colony formation assay (anchorage dependent growth) of HeLa and CaSKi cells after the addition of inhibitors for 24 hours. **D)** Colony formation assay (anchorage dependent growth) of HeLa and CaSKi cells after transfection of a pool of four specific STAT3 siRNA for 72 hours. **E)** Soft agar assay of HeLa and CaSKi cells after the addition of inhibitors for 24 hours. **F)** Soft agar assay of HeLa and CaSKi cells after transfection of a pool of four specific STAT3 siRNA for 72 hours. **G)** Representative western blot of cryptotanshinone dose response after 24 hours. **H)** Representative western blot after transfection of a pool of four specific STAT3 siRNA for 72 hours. Samples were probed with antibodies detecting total and phosphorylated STAT3, STAT3 target gene products cyclin D1 and p21 and HPV oncoproteins E6 and E7. GAPDH served as a loading control. Error bars represent the mean +/- standard deviation of a minimum of three biological repeats. **P<0.01, ***P<0.001 (Student’s t-test).

STAT3 is a key mediator of cell proliferation [52,53]. Given this information it was pertinent to investigate the consequences of inhibiting STAT3 activation in HPV positive cervical cancer cells by the application of two chemically distinct inhibitors of STAT3 (cryptotanshinone and S3I-201) or by transfecting cells with a pool of STAT3 specific siRNA [23]. Growth curves were performed with HeLa and CaSKi cells treated with DMSO or STAT3 inhibitors or transfected with scramble or STAT3 specific siRNA. Compared to controls, blockade of STAT3 activity or loss of its expression resulted in a significant reduction in cell growth over the time course of treatment (Fig 12A and 12B). Additionally, treatment with STAT3 inhibitors or depletion of STAT3 protein suppressed the ability of HeLa and CaSKi cells to form colonies under anchorage-dependent (Fig 12C and 12D) and anchorage-independent (Fig 12E and 12F) conditions. To further evaluate the impact of STAT3 inhibition, we assessed the levels of key cell cycle proteins. STAT3 phosphorylation was decreased when treated with increasing concentrations of cryptotanshinone (Fig 12G) and this correlated with a decrease in cyclin D1 expression, which we previously identified as a STAT3 target in HPV-containing primary keratinocytes [23]. Loss of cyclin D1 expression was associated with an increase in the levels of the cyclin dependent kinase inhibitor p21^WAF1/KIP1^ (Fig 12G). Changes to the complement of cell cycle control proteins as a result of STAT3 inhibition correlated with a loss in HPV oncoprotein expression in both HeLa and CaSKi cells (Fig 12G). Similar effects on cyclin D1, p21 and HPV oncoprotein expression were observed in cells treated with STAT3-specific siRNA (Fig 12H).

These observations prompted us to explore whether upstream components in the STAT3 activation pathway are also necessary for HPV positive cancer cell proliferation. To this end colony formation assays were used to measure anchorage-dependent growth of HeLa and CaSKi cells in which NFκB was inhibited either by treatment with the IKKi small molecule inhibitor or by over-expression of the transdominant IκBa protein. NFκB inhibition significantly reduced the ability of HPV positive cancer cells to form colonies (S11A Fig). Next, we tested the upstream activators of NFκB. Blockade of Rac1 activity with the NSC compound or overexpression of Rac1-DN was detrimental to HPV positive cancer cell proliferation and resulted in approximately 50% fewer colonies than the controls (S11B Fig). Finally, we assessed the contribution of AKT to HPV positive cancer cell proliferation. Loss of active AKT, either as a result of AKTi treatment or overexpression of the catalytically inactive AKT mutant, also impaired cell proliferation (S11C Fig). Similar differences in anchorage-independent growth were observed when these proteins were perturbed (S11D and S11E Fig). When compared, loss of Rac1 and NFκB activity reduced proliferation in both HeLa and CaSKi cells, whilst the impact of AKT inhibition was more pronounced in the CaSKi cells. Collectively, these observations provide evidence that the STAT3 activation pathway is crucial for HPV positive cancer cell proliferation.

### STAT3 is necessary for HPV positive cancer cell survival

**Fig 13:**
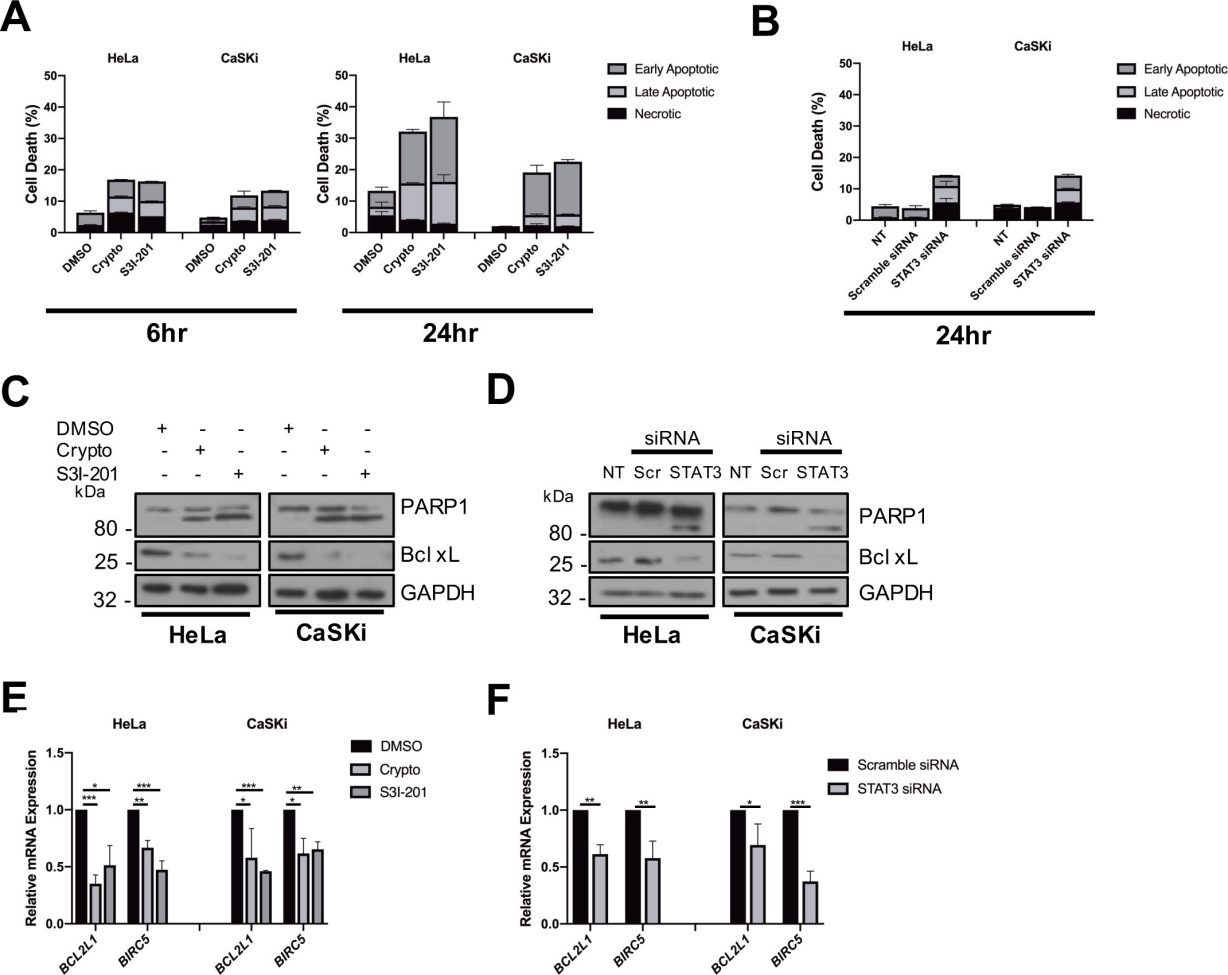
STAT3 regulates apoptosis in HPV+ cervical cancer. **A)** Flow cytometric analysis of Annexin V assay in HeLa and CaSKi cells after addition of small molecule STAT3 inhibitors for 6 and 24 hours. **B)** Flow cytometric analysis of Annexin V assay in HeLa and CaSKi cells after transfection of a pool of four specific STAT3 siRNA for 24 hours. **C)** Representative western blot of HeLa and CaSKi cells after addition of STAT3 inhibitors for 24 hours analysed for PARP cleavage and Bcl-_xL_ expression. GAPDH served as the loading control. **D)** Representative western blot of HeLa and CaSKi cells after transfection of a pool of four specific STAT3 siRNA for 24 hours analysed for PARP cleavage and Bcl-_X_L expression. GAPDH served as a loading control. **E)** Quantitative RT-qPCR analysis of HeLa and CaSKi cells after addition of STAT3 inhibitors for 24 hours for the pro-survival genes *BCL2L1* and *BIRC5*. U6 served as a loading control. **F)** Quantitative RT-qPCR analysis of HeLa and CaSKi cells after transfection of a pool of four specific STAT3 siRNA for 72 hours. Cells were analysed for the pro-survival genes *BCL2L1* and *BIRC5*. *U6* served as a loading control. Error bars represent the mean +/- standard deviation of a minimum of three biological repeats. *P<0.05, **P<0.01, ***P<0.001 (Student’s t-test).

In light of the impact of STAT3 inhibition on HPV+ cervical cancer cell proliferation, we sought to address whether the absence of active STAT3 induced apoptosis. STAT3 activity was inhibited in HeLa and CaSKi cells by treatment with STAT3 small molecule inhibitors for 6 and 24 hours (Fig 13A) or STAT3 protein depleted by transfection of a pool of STAT3-specific siRNA (Fig 13B), and the degree of phosphatidylserine exposure on the plasma membrane measured by Annexin V stain. At both 6 and 24 hours post treatment, inhibition of STAT3 or loss of STAT3 protein led to a significant increase in apoptosis compared to controls in both cell lines. We next demonstrated the activation of caspase 3 by measuring the degree of cleavage of the caspase 3 substrate poly(ADP) ribose polymerase (PARP) by western blot. As shown, inhibition of STAT3 activity (Fig 13C) or loss of STAT3 protein (Fig 13D) promoted the appearance of the faster migrating, proteolytically cleaved, form of PARP. Appearance of cleaved PARP was coincident with reduced expression of the anti-apoptotic Bcl-2 family protein Bcl-_XL_. RT-qPCR revealed the loss of survival factors such as Bcl-_XL_ (*bcl2l1*) and Survivin (*birc5*) to be at the transcriptional level (Fig 13E and 13F). Thus, STAT3 is essential for HPV+ cervical cancer cell survival.

### STAT3 and IL-6 engage in an autocrine positive feedback loop in HPV positive cervical cancer cells

STAT3 has been shown to engage in mechanisms to augment NFκB activation and maintain IL-6 expression in a number of cancers and inflammatory disorders [54–57]. To investigate if such a positive feedback loop exists in cervical cancer cells we used siRNA to deplete STAT3 from HeLa and CaSKi cells and then measured the impact on IL-6 expression (S12 Fig). Upon STAT3 depletion we observed a marked reduction in the levels of IL-6 mRNA, IL-6 protein expression and secretion, indicating that STAT3 likely drives a mechanism to maintain enhanced IL-6 expression in cervical cancer cells.

### IL-6 expression correlates with cervical disease progression

**Fig 14:**
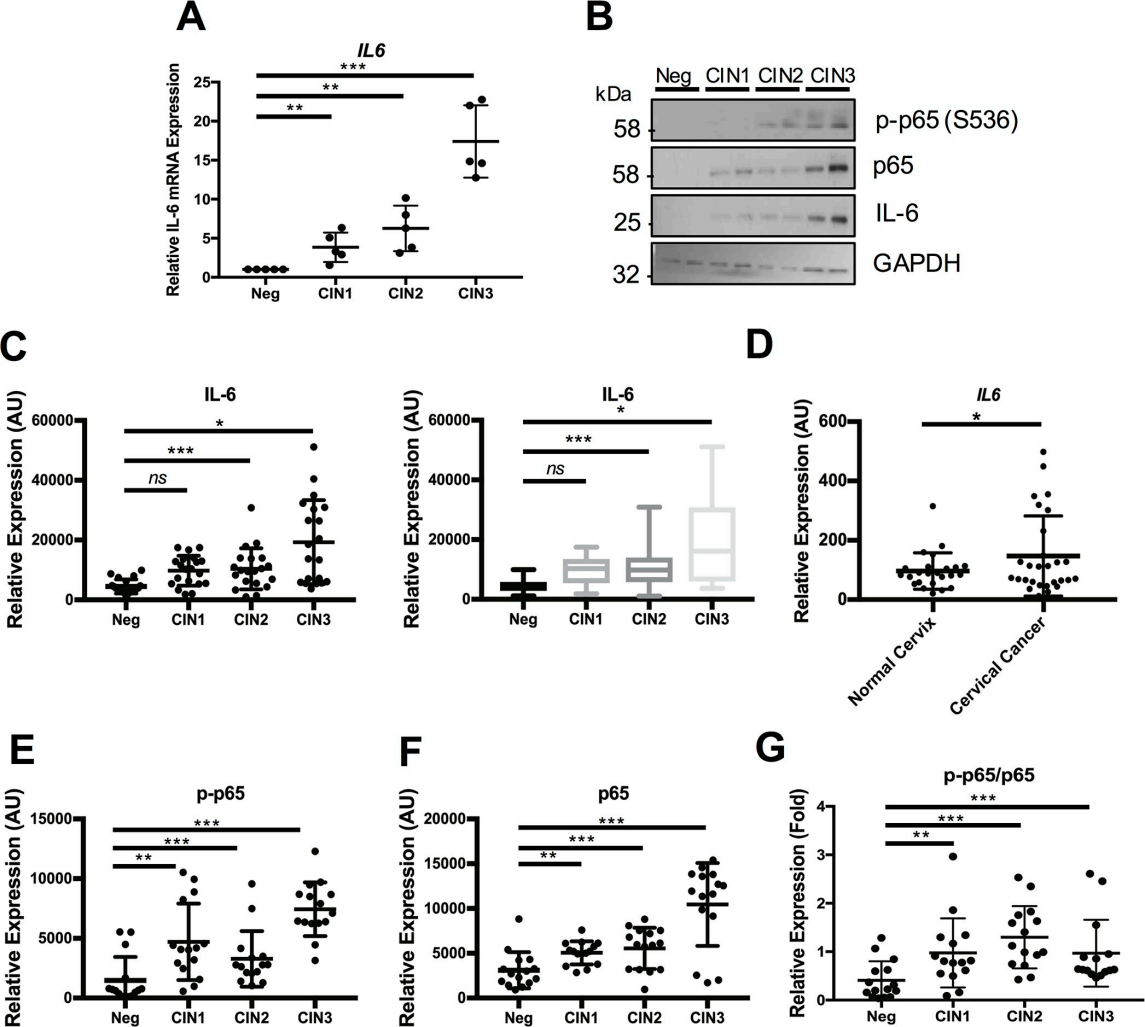
Phosphorylated p65, p65 and IL-6 expression correlate with cervical disease progression. **A)** Scatter dot plot of RT-qPCR analysis of IL-6 mRNA expression from a panel of cervical cytology samples representing CIN lesions of increasing grade. Five samples from each clinical grade (negative (Neg) and CIN I-III) were analysed and mRNA levels were normalized to the negative samples. Samples were normalized against U6 mRNA levels. Bars are the means ± standard deviation. **P<0.01, ***P<0.001 (Student’s t-test). **B)** Representative western blots of cervical cytology samples of CIN lesions of increasing grade analysed for phosphorylated and total p65 and IL-6 protein expression. GAPDH served as a loading control. **C-D)** Scatter dot plot (left) and box plot (right) of densitometry analysis of a panel of cytology samples for IL-6 expression. Twenty samples from each clinical grade (neg, CIN I-III) were analysed by western blot and densitometry analysis was performed using ImageJ. GAPDH was used as a loading control. **D)** Scatter dot plot of data acquired from the dataset GSE9750 on the GEO database. Arbitrary values for the mRNA expression of IL-6 in normal cervix (n = 23) and cervical cancer (n = 27) samples were plotted. **E-G)** Scatter dot plot of densitometry analysis of a panel of cytology samples for **E)** phosphorylated p65 and **F)** total p65. Twenty samples from each clinical grade (neg, CIN I-III) were analysed by western blot and densitometry analysis was performed using ImageJ. GAPDH was used as a loading control. **G)** Scatter dot plot of densitometry analysis of a panel of cytology samples for the ratio of phosphorylated p65 to total p65 expression. Error bars represent the mean +/- standard deviation. *P<0.05, **P<0.01, ***P<0.001 (Student’s t-test).

The IL-6—STAT3 signalling axis has emerged as a key contributor to carcinogenesis in a number of cancers [21], often resulting in increased IL-6 expression as observed in lung cancer and head and neck cancers [58,59]. We analysed cervical liquid-based cytology samples from a cohort of HPV16+ patients representing the progression of disease development (CIN1-CIN3) and compared this to HPV- normal cervical tissue to explore the role of IL-6 in cervical disease. Firstly, we observed an increase in the levels of *IL6* mRNA expression, which correlated with disease progression through CIN1-CIN3 (Fig 14A), with the greatest increase observed in CIN3 samples when compared with normal cervical tissue. Importantly, we also noted an increase in IL-6 protein levels, which correlated with disease progression (Fig 14B), again showing the largest increase in CIN3. Analysis of a larger cohort of clinical samples revealed a breadth in the levels of IL-6 protein expression, particularly in the CIN3 samples, which separated into 2 subsets; IL-6 high (n = 9; 45%) and IL-6 low (n = 11; 55%) (Fig 14C, left). To view the spread of IL-6 expression, we performed a Box and Whisker plot analysis (Fig 14C, right). The data clearly show that despite the presence of IL-6 outliers, levels of IL-6 protein expression are higher in the majority of CIN3 samples, with the IL-6 low subset still significantly higher when compared to healthy controls. To corroborate these findings in primary tumours, we mined an available microarray database of normal cervical samples against cervical cancer samples and revealed a statistically significant increase in *IL6* mRNA expression in the cervical cancer samples (Fig 14D). As with our analysis of CIN, *IL-6* expression in the cervical cancer cases clearly separates into 2 subsets; *IL-6* high (n = 7; 25.9%) and *IL-6* low (n = 20; 74.1%). As our mechanistic analysis revealed a significant role for NFκB in IL-6 production, we wished to determine whether expression and activation of this critical transcription factor was also increased in the patient samples available. Western blot analysis revealed an increase in both the levels of total and phosphorylated p65 protein that correlated with disease progression (Fig 14B). Quantification of the larger cohort of cytology samples showed that this increase was statistically significant (Fig 14E–14G). Together, these data demonstrate that p65 and IL-6 levels are increased in HPV associated cervical disease.

## Discussion

**Fig 15:**
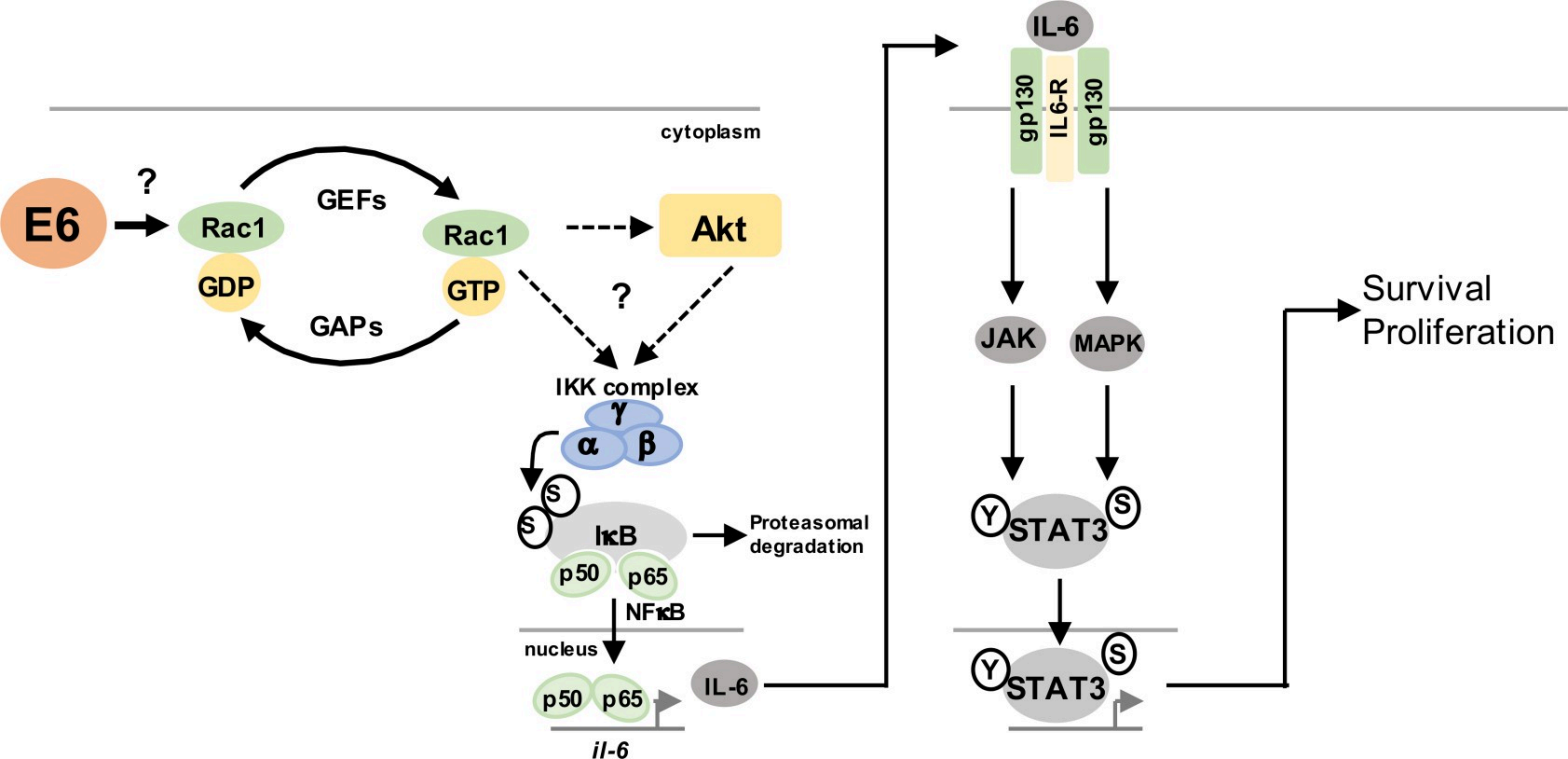
E6 activates a Rac-1/NFκB mediated STAT3 pathway in HPV+ cervical cancer. Schematic diagram of E6 mediated activation of STAT3 signalling in HPV+ cervical cancer cells. Through an undetermined mechanism E6 activates a pathway containing the Rac1 GTPase and its substrate AKT resulting in NFκB phosphorylation and IL-6 transcription. IL-6 functions in an autocrine and paracrine manner to induce STAT3 phosphorylation and nuclear translocation. Active STAT3 drives a gene expression programme required for cervical cancer cell proliferation and survival.

Oncogenic viruses can activate STAT3 to increase cell proliferation, enhance viral replication and this ultimately can contribute to tumourigenesis [22]. Previously, we used a primary cell culture model to show that the E6 oncoprotein activates STAT3 signalling in primary keratinocytes during the HPV18 lifecycle [23]. We revealed that STAT3 activation correlates with cervical disease progression. Mechanistically, we demonstrated that JAK2 and MAP kinases were responsible for phosphorylating STAT3 in HPV containing cells. Despite these advances, the host factors co-opted by E6 to drive these events were not fully explored. In this report we identified the upstream signalling pathway responsible for STAT3 phosphorylation in HPV positive cells. We reveal that E6 regulates a signalling pathway necessary for production of the cytokine IL-6. Further, we identified a key role for the small GTPase Rac1 in activating NFκB, which induced IL-6 transcription. Ultimately, we deciphered a signalling circuit critical for STAT3 activation by HPV (Fig 15).

Dysregulation of inflammatory cytokine signalling is an emerging mechanism in transformation and IL-6 is over-expressed in diverse cancers, correlating with increased STAT3 activity [21]. IL-6 displays pleiotropic functions, being both pro-inflammatory and immunosuppressive by interacting with the surrounding stroma of tumours [60]. In HNSCC and oral squamous cell carcinoma, serum levels of IL-6 are significantly higher than control patients and serum IL-6 is a potential biomarker for these cancers [61]. Additionally, targeting IL-6 in combination with EGFR inhibitors such as Cituximab is currently being investigated as a potential therapy for HNSCC due to the resistance to EGFR inhibition seen in many tumours [62,63].

In cervical cancer, IL-6 expression promotes tumour proliferation by inducing vascular epithelial growth factor (VEGF)-dependent angiogenesis in a STAT3 dependent manner [64] and has also been suggested as a potential biomarker [65]. HPV16 and HPV18 E6 oncoproteins have been demonstrated to be required for the expression and secretion of IL-6 in NSCLC cells [28]; however, the role of E6 in driving IL-6 expression in cervical cancer is unclear. Furthermore, the contribution of IL-6 to STAT3 activation in cervical cancer remains poorly defined. The increased phosphorylation of STAT3 in HPV positive cervical cancer cells was attributed to an increase in IL-6 expression by HPV E6 and the induction of autocrine/paracrine IL-6/gp130/STAT3 signalling. In cancer cells, EGFR signalling can induce STAT3 activation [66]; however, the data here identified that blockade of IL-6 or gp130 signalling using neutralising antibodies abolished STAT3 phosphorylation, suggesting that IL-6/gp130 is the major determinant for STAT3 phosphorylation in HPV+ cervical cancer cells. Interestingly, whilst anti-IL-6 treatment did reduce the levels of STAT3 activation, it was not as effective as when cells were treated with the anti-gp130 antibody. Whilst we cannot discount that this difference reflects the efficacy of the antibody reagents, an alternative explanation could be provided by the observation that HPV positive cervical cells show increased expression of a number of gp130 binding cytokines (e.g. OSM, LIF and IL-10). These may be responsible for the residual STAT3 phosphorylation observed [67,68].

We identified NFκB as an essential upstream mediator of IL-6 expression. NFκB is a key component of the inflammatory response and a key hallmark of cancer [69]. The induction of inflammation by diverse mechanisms contributes to around 20% of cancers. Previous data suggests that inflammation induced by HPV infection may contribute to HPV induced cervical cancers [70,71]. Indeed, several genes known to be induced by the inflammatory response, including COX-2 [72], are up-regulated in cervical cancer.

The role of NFκB in cervical carcinomas remains controversial, with HPV showing the potential to both activate and inhibit the transactivation function of NFκB [30,73,74]. HPV E6 has been reported to increase the expression of NFκB components and induce NFκB DNA binding activity, increasing pro-inflammatory cytokine expression [75]. Additionally, E6 can reduce the expression of the deubiquitinase CYLD, a known negative regulator of NFκB signalling, in hypoxic cells [31]. In contrast, E6 has been shown to inhibit NFκB transcriptional activity, whilst HPV E7 can attenuate p65 nuclear translocation [76]. The data presented here demonstrate that HPV18 E6 increases the phosphorylation of p65, essential for its nuclear translocation and transactivation capability. Furthermore, we demonstrate that NFκB is essential for IL-6 expression in HPV positive cervical cancer cells.

Our study uncovered a signalling circuit linking E6 to NFκB activation and IL-6 production. Chief amongst these, we found that the Rac1 GTPase was crucial in mediating the activation of NFκB. Cells expressing E6 were enriched for the GTP bound form of Rac1, indicating that E6 has the ability to activate the GTPase function of Rac1. The mechanism by which this occurs is currently not known, although it does not require interactions with E6AP, p53 or cellular PDZ domain containing proteins as classical E6 mutants deficient in these abilities are still able to increase IL-6 expression and STAT3 phosphorylation. Use of small molecule inhibitors or dominant negative forms of Rac1 support the idea that Rac1 GTPase function and interaction with downstream targets are crucial for the E6-mediated activation of NFκB. In this regard, we also identified a role for the protein kinase AKT in HPV-mediated NFκB activation. AKT can be activated by Rac1 [43] and has been shown to regulate NFκB under certain circumstances [36,77,78]. In PTEN-null cells, AKT activates NFκB through binding of the downstream components mTOR and Raptor to the IKK complex, stimulating NFκB activation [77]. Additionally, AKT can directly phosphorylate and activate IKKα at T23 to enhance p65 phosphorylation [36]. Our data demonstrate that AKT contributes to the phosphorylation of p65 and the expression on IL-6 in HPV positive cervical cancer; however, inhibition of AKT only partially reduced IL-6 expression, suggesting alternative components upstream may be involved in NFκB mediated IL-6 expression. Of particular interest, we noted that CaSKi cells appeared more sensitive to inhibition of AKT than HeLa cells. In cervical cancer, the *PIK3CA* gene is extensively mutated, with the most common mutation (E545K) resulting in constitutive PI3K/AKT signalling [79]. This oncogenic mutation can activate IKK/NFκB signalling and increase IL-6 secretion and paracrine STAT3 activation in epithelial cells [37]. Interestingly, whilst HeLa cells have wild type *PIK3CA*, CaSKi cells encode the E545K mutant [80]. It may therefore be possible that in cells with constitutive PI3K/AKT signalling, AKT inhibition has a greater contribution to the NFκB/IL-6 signalling axis than in cells expressing wild type *PIK3CA* [80].

Loss of active STAT3 had a significant impact on both cervical cancer cell proliferation and survival. The increased apoptosis observed was coupled with a loss of expression of key pro-survival factors such as Bcl-_XL_ and Survivin, reinforcing the concept that STAT3 inhibition is deleterious to HPV cancer cell survival [24,25]. However, it was imperative to obtain a more comprehensive understanding of the host pathways necessary for STAT3 activation. Interestingly, targeting of key upstream factors necessary for the autocrine activation of STAT3 also impaired proliferation indicating that this signalling circuit is essential in cervical cancer cells.

The NFκB–IL-6—STAT3 signalling axis is important to cancer biology. We previously demonstrated increased STAT3 phosphorylation in cervical disease [23] and we now demonstrate a similar link between IL-6 levels and cervical disease. We noted increased IL-6 mRNA expression in high-grade cervical disease samples and in cervical cancer samples compared to healthy controls. Interestingly, we observed that IL-6 protein expression in CIN3 and cervical cancer clearly stratifies into two sub-populations; IL-6 high and IL-6 low. These findings suggest there may be diversity in the requirement for IL-6 in HPV positive cervical cancers. As STAT3 can be activated by several soluble factors in cervical cancer cells, such as OSM [67], EGF [81] and IL-10 [82], it is possible that the IL-6 low cancers might be dependent on these additional cytokines to maintain active STAT3. Furthermore, IL-6 independent STAT3 activation can be found in other cancers, including head and neck cancers and haemopoietic cancers [83–85], suggesting that not all cancers that have high STAT3 activity necessarily have high IL-6 expression. In diffuse large B-cell lymphoma (DLBCL), a subgroup called the activated B cell–like (ABC) DLBCL, are characterised by high IL-6 expression and STAT3 activity and, importantly, are selectively sensitive to JAK inhibition when compared to germinal center B-cell (GCB) DLBCL [86]. Our data demonstrate that although IL-6 may be essential for the activation of STAT3 in our cervical cancer cell culture system, the heterogeneity of IL-6 expression observed in clinical samples of cervical disease and cervical cancers warrants further investigation to allow the proper stratification of potential therapeutics targeting this pathway.

The data presented here demonstrate that NFκB is essential for the induction of IL-6 and the autocrine/paracrine induction of STAT3 phosphorylation in HPV+ cervical cancer cells. We identify that the Rac1 GTPase and protein kinase AKT lie upstream of NFκB/IL-6 signalling. Although therapies are not currently available, strategies to target the NFκB–IL-6 –STAT3 signalling axis may benefit the treatment of HPV+ cancers.

## Methods and materials

### Cervical cytology samples

Cervical cytology samples were obtained from the Scottish HPV Archive (http://www.shine/mvm.ed.ac.uk/archive.shtml), a biobank of over 20,000 samples designed to facilitate HPV associated research. The East of Scotland Research Ethics Service has given generic approval to the Scottish HPV Archive as a Research Tissue Bank (REC Ref 11/AL/0174) for HPV related research on anonymised archive samples. Samples are available for the present project though application to the Archive Steering Committee (HPV Archive Application Ref 0034). RNA and protein were extracted from the samples using Trizol as described by the manufacturer (ThermoFischer Scientific, USA) and analysed as described.

### Cell culture

C33A (HPV negative cervical carcinoma), DoTc2 4510 (HPV negative cervical carcinoma), SiHa (HPV16 positive cervical squamous carcinoma), CaSKi (HPV16 positive cervical squamous carcinoma), SW756 (HPV18 positive squamous carcinoma) and HeLa (HPV18 positive cervical epithelial adenocarcinoma) cells were purchased from ATCC and grown in Dulbecco’s modified Eagle’s media (DMEM) supplemented with 10% Foetal Bovine Serum (FBS) (ThermoFischer Scientific, USA) and 50 U/ml penicillin/streptomycin (Lonza). NHK cells were purchased from Lonza and maintained as described [7].

### Inhibitors and cytokines

The IKKα/β inhibitor IKK inhibitor VII was purchased from Calbiochem and used at a final concentration of 5 μM unless otherwise stated. The AKT1/2 inhibitor AKT VIII was purchased from Calbiochem and used at a final concentration of 5 μM unless otherwise stated. Recombinant human IL-6 was purchased from R&D Systems and used at a final concentration of 20 ng/mL unless otherwise stated. Recombinant TNFα was purchased from PeproTech EC Ltd and used at a final concentration of 10 ng/mL. All compounds were used at concentrations required to minimise potential off-target activity. Neutralising IL-6 antibody (ab6628) was purchased from Abcam and used at a final dilution of 1:400. Neutralising gp130 antibody (MAB228) was purchased from R&D Systems and used at a final concentration of 1 μg/mL.

### Plasmids and siRNAs

Plasmids expressing HPV18 E6 sequences were amplified from the HPV18 genome and cloned into peGFP-C1 with *Sal*I and *Xma*I restriction enzymes [23]. The plasmid driving Firefly luciferase from the IL-6 promoter was a kind gift from Prof Derek Mann (University of Newcastle) and used as previously described [87]; the ConA promoter (that contains tandem NFκB response elements) [88] and a constitutive Renilla luciferase reporter (pRLTK) were previously described [87]. The IκBα S33/36A mutant was a kind gift from Prof Ronald Hay (University of Dundee). pLNCX myr HA AKT1 K179M (AKT-DN) vector was purchased from Addgene (Addgene, 9006) and murine retrovirus envelope and GAG/polymerase plasmids were kindly provided by Professor Greg Towers (University College London). pcDNA-GFP-Rac1-T17N (Rac1-DN) was kindly provided by Professor Adrian Whitehouse (University of Leeds). MSCV-HA-HPV18 E6 was kindly provided by Dr Elizabeth White (University of Pennsylvania). For siRNA experiments, two siRNA sequences specifically targeting HPV18 E6 were purchased from GE Healthcare with the following sequences: ^5’^CUAACACUGGGUUAUACAA‘3 and 5’CTAACTAACACTGGGTTAT^‘3^. For HPV16, a single siRNA targeting the HPV16 E6 protein was purchased from Santa Cruz Biotechnology (SCBT; sc-156008). For each experiment, 40 nM of pooled siRNA was used and cell lysates were harvested after 72 hours.

### Transfections and mammalian cell lysis

Transient transfections were performed with a DNA to Lipofectamine 2000 (ThermoFischer) ratio of 1:2.5. 48 h post transfection, cells were lysed in Leeds lysis buffer for western blot [88].

### Colony formation assay

48 hr post-transfection, cells were trypsinised and reseeded in a six well plate at 500 cells per well and left to incubate for 14–21 days. Colonies were then stained (1% crystal violet, 25% methanol) and colonies were counted manually. Each experiment was repeated a minimum of 3 times.

### Soft agar assay

Cells were transfected as required. 60 mm dishes were coated with a layer of 1% agarose (ThermoFischer Scientific, USA) in 2X DMEM (ThermoFischer Scientific, USA) supplemented with 20% FBS. 48 hr post-transfection, cells were trypsinised and added to 0.7% agarose in 2X DMEM (ThermoFischer Scientific, USA) supplemented with 20% FBS at 1000 cells/mL. Once set, DMEM supplemented with 10% FBS and 50 U/mL penicillin was added. The plates were then incubated for 14–21 days. Each experiment was repeated at least three times unless stated otherwise. Visible colonies were counted manually.

### Annexin V assay

Annexin V apoptosis assay (TACS Annexin V kit; 4830-250-K) was performed as indicated on the product datasheet. Briefly, cells were seeded in 6 well plates at a density of 1 x 10^6^ cells/mL and were treated as required per experiment. Cells were then trypsinised and collected by centrifugation at 700 x g for 5 mins. Cells were then washed in cold PBS and re-centrifuged. 1x10^6^ cells were then incubated in 100 μL Annexin V reagent (10 μL 10 x binding buffer, 10 μL propidium iodide, 1 μL Annexin V-FITC (diluted 1 in 500) and 880 μL ddH_2_O) for 15 mins at room temperature in the dark. 400 μL of 1 x binding buffer was then added before analysis by flow cytometry. Samples were processed on an LSRFortessaTM cell analyzer (BD) and the PI histograms analysed on modfit software.

### Western blotting

Total protein was resolved by SDS-PAGE (10–15% Tris-Glycine), transferred onto Hybond nitrocellulose membrane (Amersham biosciences) and probed with antibodies specific for phospho-STAT3 (S727) (ab32143, abcam), phospho-STAT3 (Y705) (9131, Cell Signalling Technology (CST)), STAT3 (124H6: 9139, CST), phospho-NFκB p65 (S536) (93H1; 3033, CST), NFκB p65 (D14E12; 8242, CST), phospho-AKT (T308) (244F9; 4056, CST), phospho-AKT (S473) (D9E; 4060, CST), AKT (9272, CST), IL-6 (ab6672, abcam), HA (HA-7, Sigma H9658), GFP (B-2: sc-9996, SCBT), FLAG (F3165, Sigma), GAPDH (G-9, SCBT), PARP-1 (9542, CST) and Bcl-_xL_ (2764, CST). Western blots were visualized with species-specific HRP conjugated secondary antibodies (Sigma) and ECL (Thermo/Pierce). Densitometry analysis was performed using ImageJ analysis software (NIH, USA).

### Retrovirus transduction

pLNCX AKT vector (Addgene, 9006) and MSCV-HA-18E6 were transfected into HEK293TT cells with murine retrovirus envelope and GAG/polymerase plasmids (kindly provided by Professor Greg Towers, University College London) using PEI transfection reagent as previously described [23]. After 48 hours the media was removed from the HEK293TT cells and added to HeLa cells for 16 hours. After this time, the virus was removed and replaced with DMEM and cells were harvested 48 hours after transduction.

### Quantitative real-time PCR

Total RNA was extracted using the E.Z.N.A. Total RNA Kit I (Omega Bio- Tek) according to the manufacture’s protocol. One μg of total RNA was DNase treated following the RQ1 RNase-Free DNase protocol (Promega) and then reverse transcribed with a mixture of random primers and oligo(dT) primers using the qScriptTM cDNA SuperMix (Quanta Biosciences) according to instructions. RT- qPCR was performed using the QuantiFast SYBR Green PCR kit (Qiagen). The PCR reaction was conducted on a Corbett Rotor-Gene 6000 (Qiagen) as follows: initial activation step for 10 min at 95°C and a three-step cycle of denaturation (10 sec at 95°C), annealing (15 sec at 60°C) and extension (20 sec at 72°C) which was repeated 40 times and concluded by melting curve analysis. The data obtained was analysed according to the ΔΔCt method using the Rotor-Gene 6000 software [89]. Specific primers were used for each gene analysed. U6 served as normaliser gene.

### Luciferase reporter assays

Cells were seeded into 12 well dishes and transfected the following day using PEI with reporter plasmids expressing firefly luciferase under the control of the *IL-6* promoter or the *ConA* promoter, which contains tandem repeats of a κB-response element [87,90]. Where appropriate, cells were co-transfected with plasmids expressing GFP or GFP-E6. To normalise for transfection efficiency, pRLTK Renilla luciferase reporter plasmid was added to each transfection. After 24 hours, samples were lysed in passive lysis buffer (Promega) and activity measured using a dual-luciferase reporter assay system (Promega) as described [91].

### Immunofluorescent staining

Cells were seeded onto coverslips and, 24 hr later, were transfected as required. 24 hr after transfection, cells were fixed with 4% paraformaldehyde for 10 min and then permeabilised with 0.1% (v/v) Triton for 15 minutes. Cells were then incubated in primary antibodies in PBS with 4% BSA overnight at 4°C. Primary antibodies were used at a concentration of 1:400. Cells were washed thoroughly in PBS and then incubated with Alex-fluor conjugated secondary antibodies 594 and Alexa 488 (1:1000) (Invitrogen) in PBS with 4% BSA for 2 hours. DAPI was used to visualise nuclei. Coverslips were mounted onto slides with Prolong Gold (Invitrogen). Quantification of nuclear localisation was quantified as described [92].

### ELISA

The human IL-6 DuoSet® ELISA was purchased from R&D Systems and was used according to the manufacturer’s instructions.

### Rac1 activation assay

The activation of Rac1 was determined by pulldown assay as previously described [51] and following the manufacturer’s instructions (Cell Biolabs). Cell lysates were incubated with PAK PBD agarose beads, which have a high affinity for GTP-Rac1. Affinity precipitated activated Rac1-GTP levels were then analysed by immunoblotting using a Rac1 specific antibody (Cell Biolabs).

### Microarray analysis

For microarray analysis, a dataset of 27 cervical cancer cases and 23 normal cervix samples was utilised. Microarray data was obtained from GEO database accession number GSE9750.

### Statistical analysis

Where indicated, data was analysed using a two-tailed, unpaired Student’s t-test.

## Supporting information

S1 Fig HPV18 E6 induces IL-6 expression in primary keratinocytes. **A)** Normal human keratinocytes (NHK) were transfected with GFP or GFP tagged HPV18 E6 and analysed for IL-6 mRNA expression by RT-qPCR. Samples were normalized against U6 mRNA levels. **B)** Representative western blot of NHK cells transfected with GFP or GFP tagged HPV18 E6 and analysed for the expression of IL-6 protein. Expression of GFP was confirmed with an antibody against GFP whilst the GFP E6 fusion was detected using a HPV18 E6 antibody. GAPDH served as a loading control. **C)** NHK cells were transfected with GFP or GFP tagged HPV18 E6. The culture medium was analysed for IL-6 protein by ELISA. Data are representative of at least three biological independent repeats. Error bars represent the mean +/- standard deviation of a minimum of three biological repeats. *P<0.05, (Student’s t-test).
(TIFF)

S2 Fig HPV16 E6 induces IL-6 expression. **A)** CaSKi cells were transfected with HPV16 E6 specific siRNA and analysed for IL-6 mRNA expression by RT-qPCR. Samples were normalized against U6 mRNA levels. **B)** Representative western blot of CaSKi cells transfected with a pool of two specific siRNAs against HPV16 E6 and analysed for the expression of IL-6. Knockdown of HPV16 E6 was confirmed using antibodies against HPV16 E6 and p53. GAPDH served as a loading control. **C)** CaSKi cells were transfected with a pool of two specific siRNAs against HPV16 E6. The culture medium was analysed for IL-6 protein by ELISA. Data are representative of at least three biological independent repeats. Error bars represent the mean +/- standard deviation of a minimum of three biological repeats. *P<0.05, ***P<0.001 (Student’s t-test).
(TIFF)

S3 Fig The p53, E6AP and PDZ domain binding properties of E6 are not required for induction of IL-6 expression in cervical cells. **A)** C33A cells were transfected with GFP, GFP tagged HPV18 E6 wildtype, HPV18 E6 ΔPDZ, HPV18 E6 F4V and HPV18 L52A. Lysates were probed with antibodies against IL-6 and GAPDH served as a loading control. Expression of the GFP E6 fusions was confirmed by anti-GFP western blot and p53 western blot validated the inability of the F4V and L52A mutants to degrade p53.
(TIFF)

S4 Fig Activation of NFκB by TNF⍺ induces IL-6 expression and STAT3 nuclear translocation. **A)** Representative western blot of C33A cells treated with 20 ng/mL recombinant human TNF**⍺** for the indicated time points. Cell lysates were analysed for phosphorylated and total p65, phosphorylated and total STAT3 and IL-6 expression. GAPDH served as a loading control. Data are representative of at least three biological independent repeats. **B)** C33A cells treated with 20 ng/mL recombinant human TNF**⍺** for 60 mins were fixed and were analysed by immunofluorescence staining for total STAT3 (green) and total p65 (red) and counterstained with DAPI to highlight the nuclei (blue in the merged panels). Scale bar 20 μm.
(TIFF)

S5 Fig NFκB is required for STAT3 activity in HPV16 positive cervical cancer cells. **A)** Representative western blot of CaSKi cells treated with increasing doses of IKKi. Cell lysates were analysed for the expression of phosphorylated and total p65, phosphorylated and total STAT3 and IL-6 expression. GAPDH served as a loading control. **B)** Representative western blot of CaSKi cells transfected with mutant IκB (IκBm). Cell lysates were analysed as in **A).**
(TIFF)

S6 Fig Quantification of nuclear STAT3 from Fig 7. **A)** Scatter dot plot of percentage nuclear STAT3 from **Fig** 7E. Data represents the percentage nuclear localisation of STAT3 from 15 cells from three independent experiments. Nuclear localisation was calculated using ImageJ [92]. **B)** Scatter dot plot of percentage nuclear STAT3 from **Fig** 7F. Data represents the percentage nuclear localisation of STAT3 from 15 cells from three independent experiments. Nuclear localisation was calculated using ImageJ [92]. Error bars represent the mean +/- standard deviation. NS = not significant, ***P<0.001 (Student’s t-test).
(TIFF)

S7 Fig AKT is required for STAT3 activity in HPV16 positive cervical cancer cells. **A)** Representative western blot of CaSKi cells treated with increasing doses of the AKTi. Cell lysates were analysed for the levels of phosphorylated and total AKT and STAT3 and IL-6 protein. GAPDH served as a loading control. **B)** Representative western blot of CaSKi cells transfected with dominant negative AKT (AKT-DN). Cell lysates were analysed as in **A).**
(TIFF)

S8 Fig Quantification of nuclear STAT3 from Fig 9. **A)** Scatter dot plot of percentage nuclear STAT3 from **Fig** 9E. Data represents the percentage nuclear localisation of STAT3 from 15 cells from three independent experiments. Nuclear localisation was calculated using ImageJ [92]. **B)** Scatter dot plot of percentage nuclear STAT3 from **Fig** 9F. Data represents the percentage nuclear localisation of STAT3 from 15 cells from three independent experiments. Nuclear localisation was calculated using ImageJ [92]. Error bars represent the mean +/- standard deviation. NS = not significant, ***P<0.001 (Student’s t-test).
(TIFF)

S9 Fig Rac1 is required for STAT3 activity in HPV16 positive cervical cancer cells. **A)** Representative western blot of CaSKi cells treated with increasing doses of NSC. Cell lysates were analysed for the levels of phosphorylated and total STAT3 and IL-6 protein expression. GAPDH served as a loading control. **B)** Representative western blot of CaSKi cells transfected with Rac1 N17 (Rac1-DN). Cell lysates were analysed as in **A).**
(TIFF)

S10 Fig Quantification of nuclear STAT3 from Fig 11. **A)** Scatter dot plot of percentage nuclear STAT3 from **Fig** 11E. Data represents the percentage nuclear localisation of STAT3 from 15 cells from three independent experiments. Nuclear localisation was calculated using ImageJ [92]. **B)** Scatter dot plot of percentage nuclear STAT3 from **Fig** 11F. Data represents the percentage nuclear localisation of STAT3 from 15 cells from three independent experiments. Nuclear localisation was calculated using ImageJ [92]. Error bars represent the mean +/- standard deviation. NS = not significant, ***P<0.001 (Student’s t-test).
(TIFF)

S11 Fig NFκB, AKT and Rac1 contribute to the proliferation of HPV positive cervical cancer cells. **A)** Colony formation assay (anchorage dependent growth) of HeLa and CaSKi cells after treatment with IKKi or transfection with IkBm. **B)** Colony formation assay (anchorage dependent growth) of HeLa and CaSKi cells after treatment with AKTi or transfection with AKT-DN. **C)** Colony formation assay (anchorage dependent growth) of HeLa and CaSKi cells after treatment with NSC or transfection with Rac1-DN. **D)** Soft agar assay (anchorage independent growth) of HeLa cells after treatment with the indicated inhibitors or transfection with the indicated constructs. **E)** Soft agar assay of CaSKi cells after treatment with the indicated inhibitors or transfection with the indicated constructs. Error bars represent the mean +/- standard deviation from the appropriate control (DMSO or pcDNA) of a minimum of three biological repeats. *P<0.05, **P<0.01, ***P<0.001 (Student’s t-test).
(TIFF)

S12 Fig STAT3 contributes to IL-6 expression in HPV positive cervical cancer cells. **A)** HeLa and CaSKi cells were transfected with a pool of four specific siRNAs against STAT3 and analysed for IL-6 mRNA expression by RT-qPCR. Samples were normalized against U6 mRNA levels. **B)** Representative western blot of HeLa and CaSKi cells transfected with a pool of four specific siRNAs against STAT3. Cell lysates were analysed for total STAT3 and IL-6. GAPDH served as a loading control. **C)** HeLa and CaSKi cells were transfected with a pool of four specific siRNAs against STAT3. The culture medium was then analysed for IL-6 expression by ELISA. Data are representative of at least three biological independent repeats. Error bars represent the mean +/- standard deviation. *P<0.05, **P<0.01, ***P<0.001 (Student’s t-test).
(TIFF)
