# Supplementary figures and images for: Autocrine STAT3 activation in HPV positive cervical cancer through a virus-driven Rac1—NFκB—IL-6 signalling axis
Source: PLoS Pathog. 2019 Jun 21;15(6):e1007835. doi: 10.1371/journal.ppat.1007835 (PMC6608985; doi:10.1371/journal.ppat.1007835)

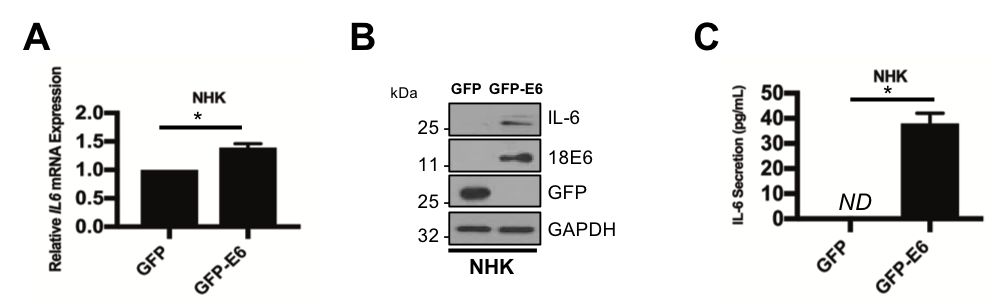

Supplement: S1 Fig — A) Normal human keratinocytes (NHK) were transfected with GFP or GFP tagged HPV18 E6 and analysed for IL-6 mRNA expression by RT-qPCR. Samples were normalized against U6 mRNA levels. B) Representative western blot of NHK cells transfected with GFP or GFP tagged HPV18 E6 and analysed for the expression of IL-6 protein. Expression of GFP was confirmed with an antibody against GFP whilst the GFP E6 fusion was detected using a HPV18 E6 antibody. GAPDH served as a loading control. C) NHK cells were transfected with GFP or GFP tagged HPV18 E6. The culture medium was analysed for IL-6 protein by ELISA. Data are representative of at least three biological independent repeats. Error bars represent the mean +/- standard deviation of a minimum of three biological repeats. *P<0.05, (Student’s t-test). (TIFF) [file ppat.1007835.s001.tiff]

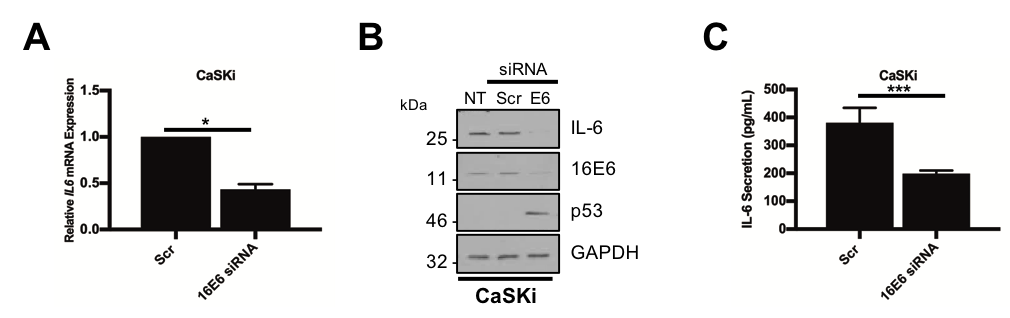

Supplement: S2 Fig — A) CaSKi cells were transfected with HPV16 E6 specific siRNA and analysed for IL-6 mRNA expression by RT-qPCR. Samples were normalized against U6 mRNA levels. B) Representative western blot of CaSKi cells transfected with a pool of two specific siRNAs against HPV16 E6 and analysed for the expression of IL-6. Knockdown of HPV16 E6 was confirmed using antibodies against HPV16 E6 and p53. GAPDH served as a loading control. C) CaSKi cells were transfected with a pool of two specific siRNAs against HPV16 E6. The culture medium was analysed for IL-6 protein by ELISA. Data are representative of at least three biological independent repeats. Error bars represent the mean +/- standard deviation of a minimum of three biological repeats. *P<0.05, ***P<0.001 (Student’s t-test). (TIFF) [file ppat.1007835.s002.tiff]

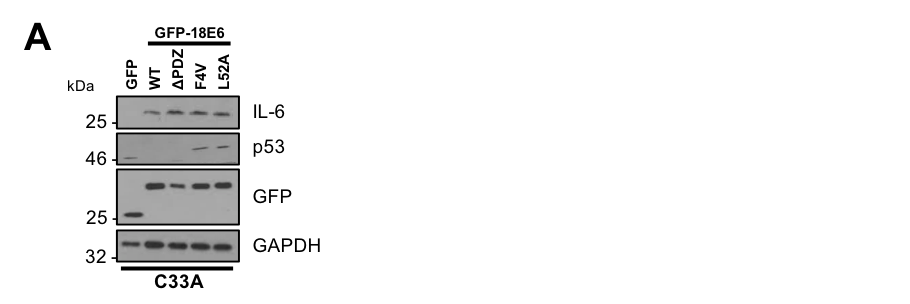

Supplement: S3 Fig — A) C33A cells were transfected with GFP, GFP tagged HPV18 E6 wildtype, HPV18 E6 ΔPDZ, HPV18 E6 F4V and HPV18 L52A. Lysates were probed with antibodies against IL-6 and GAPDH served as a loading control. Expression of the GFP E6 fusions was confirmed by anti-GFP western blot and p53 western blot validated the inability of the F4V and L52A mutants to degrade p53. (TIFF) [file ppat.1007835.s003.tiff]

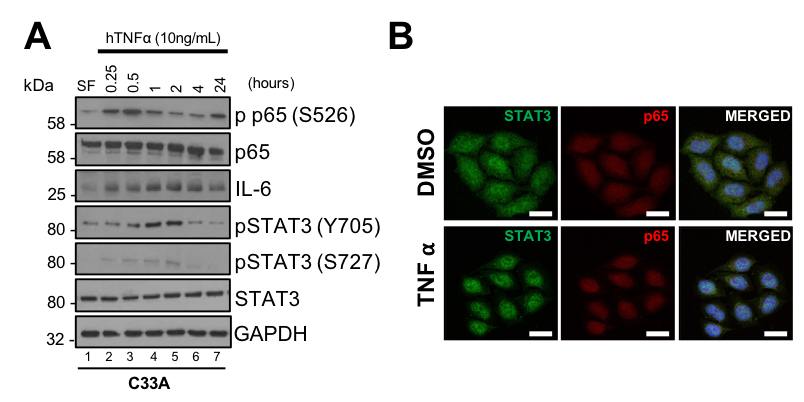

Supplement: S4 Fig — A) Representative western blot of C33A cells treated with 20 ng/mL recombinant human TNF⍺ for the indicated time points. Cell lysates were analysed for phosphorylated and total p65, phosphorylated and total STAT3 and IL-6 expression. GAPDH served as a loading control. Data are representative of at least three biological independent repeats. B) C33A cells treated with 20 ng/mL recombinant human TNF⍺ for 60 mins were fixed and were analysed by immunofluorescence staining for total STAT3 (green) and total p65 (red) and counterstained with DAPI to highlight the nuclei (blue in the merged panels). Scale bar 20 μm. (TIFF) [file ppat.1007835.s004.tiff]

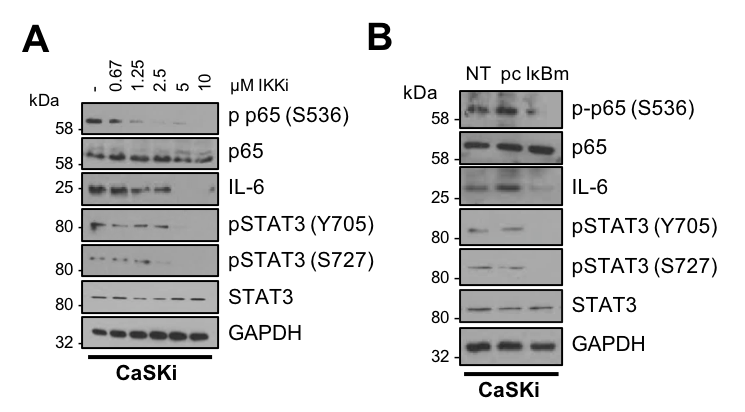

Supplement: S5 Fig — A) Representative western blot of CaSKi cells treated with increasing doses of IKKi. Cell lysates were analysed for the expression of phosphorylated and total p65, phosphorylated and total STAT3 and IL-6 expression. GAPDH served as a loading control. B) Representative western blot of CaSKi cells transfected with mutant IκB (IκBm). Cell lysates were analysed as in A). (TIFF) [file ppat.1007835.s005.tiff]

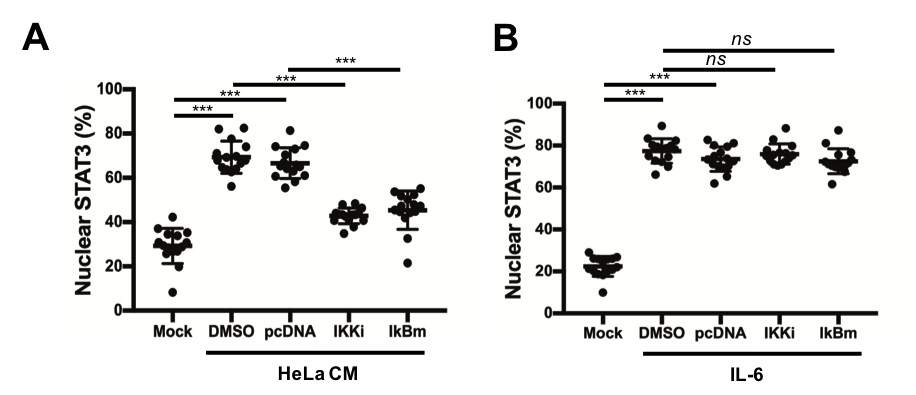

Supplement: S6 Fig — A) Scatter dot plot of percentage nuclear STAT3 from Fig 7E. Data represents the percentage nuclear localisation of STAT3 from 15 cells from three independent experiments. Nuclear localisation was calculated using ImageJ [92]. B) Scatter dot plot of percentage nuclear STAT3 from Fig 7F. Data represents the percentage nuclear localisation of STAT3 from 15 cells from three independent experiments. Nuclear localisation was calculated using ImageJ [92]. Error bars represent the mean +/- standard deviation. NS = not significant, ***P<0.001 (Student’s t-test). (TIFF) [file ppat.1007835.s006.tiff]

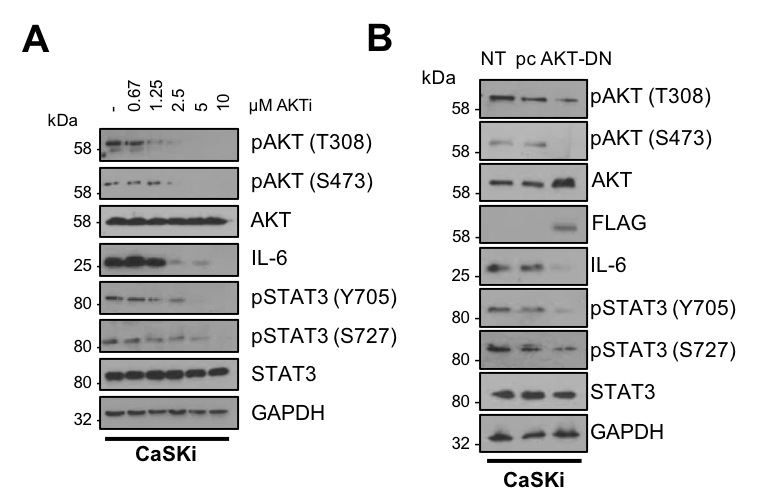

Supplement: S7 Fig — A) Representative western blot of CaSKi cells treated with increasing doses of the AKTi. Cell lysates were analysed for the levels of phosphorylated and total AKT and STAT3 and IL-6 protein. GAPDH served as a loading control. B) Representative western blot of CaSKi cells transfected with dominant negative AKT (AKT-DN). Cell lysates were analysed as in A). (TIFF) [file ppat.1007835.s007.tiff]

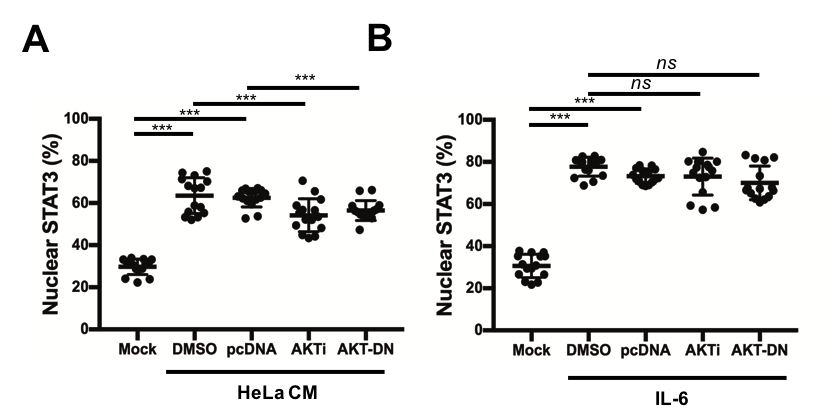

Supplement: S8 Fig — A) Scatter dot plot of percentage nuclear STAT3 from Fig 9E. Data represents the percentage nuclear localisation of STAT3 from 15 cells from three independent experiments. Nuclear localisation was calculated using ImageJ [92]. B) Scatter dot plot of percentage nuclear STAT3 from Fig 9F. Data represents the percentage nuclear localisation of STAT3 from 15 cells from three independent experiments. Nuclear localisation was calculated using ImageJ [92]. Error bars represent the mean +/- standard deviation. NS = not significant, ***P<0.001 (Student’s t-test). (TIFF) [file ppat.1007835.s008.tiff]

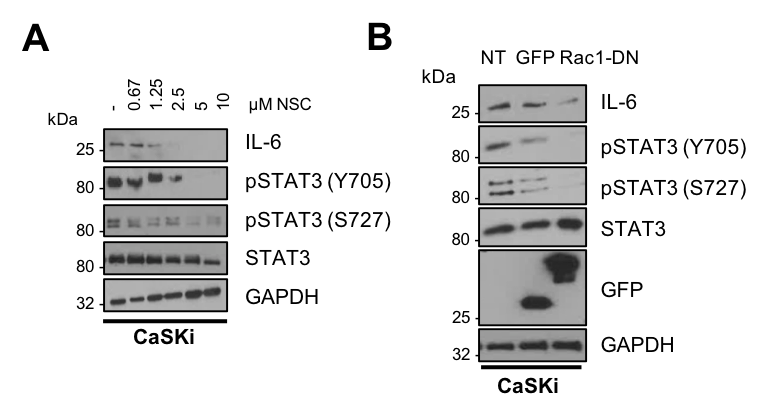

Supplement: S9 Fig — A) Representative western blot of CaSKi cells treated with increasing doses of NSC. Cell lysates were analysed for the levels of phosphorylated and total STAT3 and IL-6 protein expression. GAPDH served as a loading control. B) Representative western blot of CaSKi cells transfected with Rac1 N17 (Rac1-DN). Cell lysates were analysed as in A). (TIFF) [file ppat.1007835.s009.tiff]

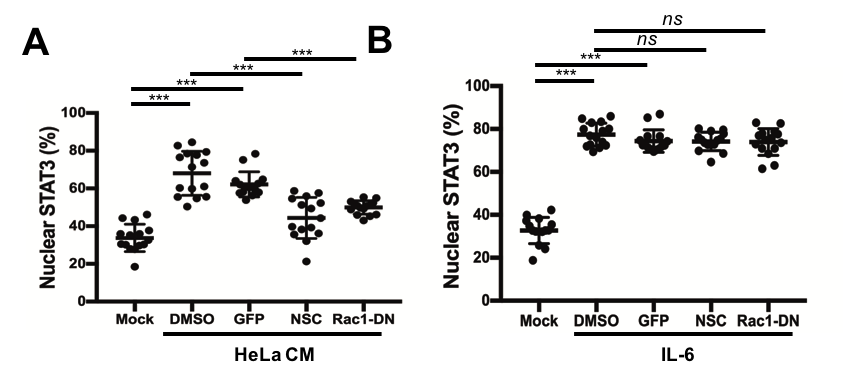

Supplement: S10 Fig — A) Scatter dot plot of percentage nuclear STAT3 from Fig 11E. Data represents the percentage nuclear localisation of STAT3 from 15 cells from three independent experiments. Nuclear localisation was calculated using ImageJ [92]. B) Scatter dot plot of percentage nuclear STAT3 from Fig 11F. Data represents the percentage nuclear localisation of STAT3 from 15 cells from three independent experiments. Nuclear localisation was calculated using ImageJ [92]. Error bars represent the mean +/- standard deviation. NS = not significant, ***P<0.001 (Student’s t-test). (TIFF) [file ppat.1007835.s010.tiff]

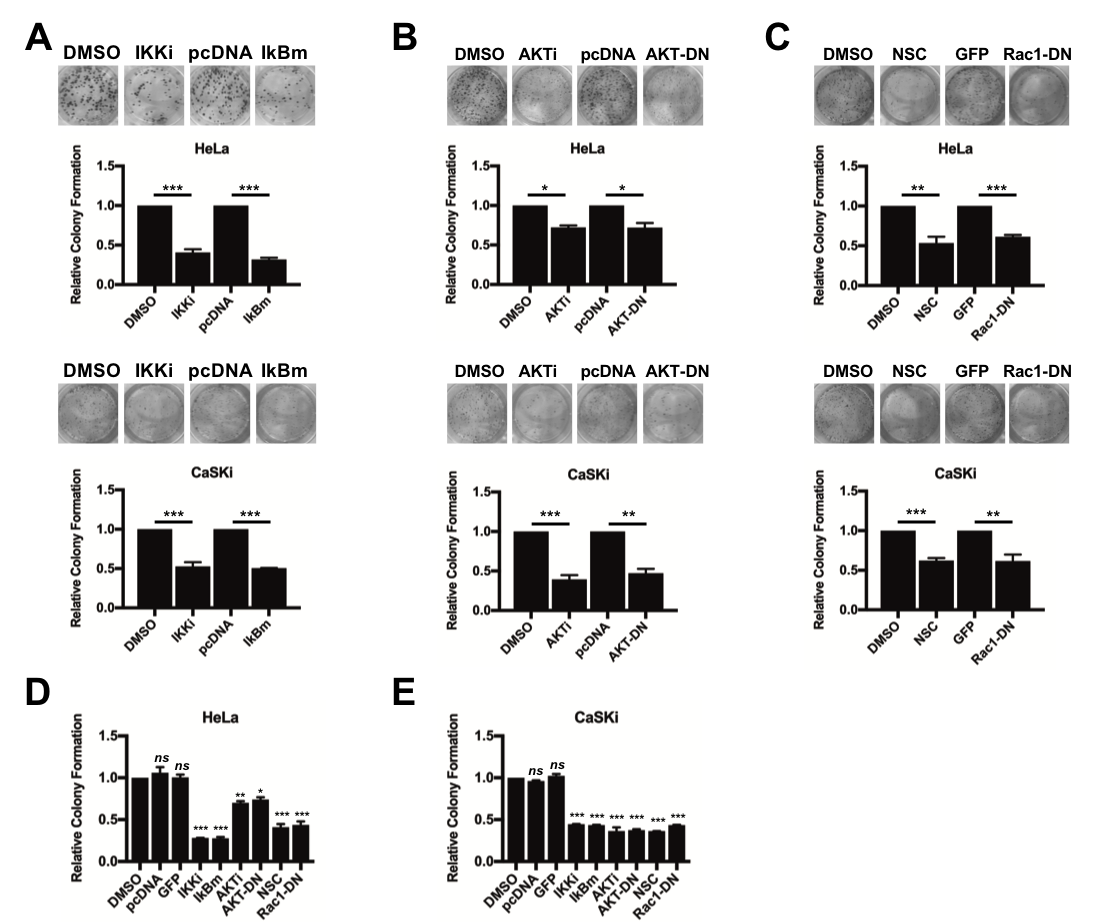

Supplement: S11 Fig — A) Colony formation assay (anchorage dependent growth) of HeLa and CaSKi cells after treatment with IKKi or transfection with IkBm. B) Colony formation assay (anchorage dependent growth) of HeLa and CaSKi cells after treatment with AKTi or transfection with AKT-DN. C) Colony formation assay (anchorage dependent growth) of HeLa and CaSKi cells after treatment with NSC or transfection with Rac1-DN. D) Soft agar assay (anchorage independent growth) of HeLa cells after treatment with the indicated inhibitors or transfection with the indicated constructs. E) Soft agar assay of CaSKi cells after treatment with the indicated inhibitors or transfection with the indicated constructs. Error bars represent the mean +/- standard deviation from the appropriate control (DMSO or pcDNA) of a minimum of three biological repeats. *P<0.05, **P<0.01, ***P<0.001 (Student’s t-test). (TIFF) [file ppat.1007835.s011.tiff]

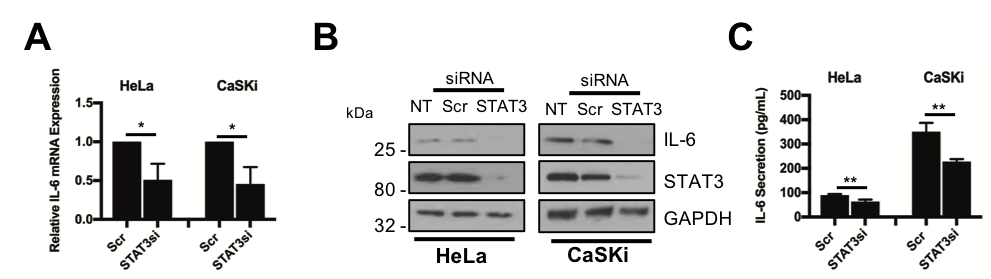

Supplement: S12 Fig — A) HeLa and CaSKi cells were transfected with a pool of four specific siRNAs against STAT3 and analysed for IL-6 mRNA expression by RT-qPCR. Samples were normalized against U6 mRNA levels. B) Representative western blot of HeLa and CaSKi cells transfected with a pool of four specific siRNAs against STAT3. Cell lysates were analysed for total STAT3 and IL-6. GAPDH served as a loading control. C) HeLa and CaSKi cells were transfected with a pool of four specific siRNAs against STAT3. The culture medium was then analysed for IL-6 expression by ELISA. Data are representative of at least three biological independent repeats. Error bars represent the mean +/- standard deviation. *P<0.05, **P<0.01, ***P<0.001 (Student’s t-test). (TIFF) [file ppat.1007835.s012.tiff]
